# Impacts of stress and aging on spore health in *Schizosaccharomyces pombe*

**DOI:** 10.1128/spectrum.01710-25

**Published:** 2025-11-07

**Authors:** Nicole L. Nuckolls, Michael T. Eickbush, Jeffrey J. Lange, Christopher J. Wood, Stephanie H. Nowotarski, Sarah E. Zanders

**Affiliations:** 1 Stowers Institute for Medical Research 4200, Kansas City, Missouri, USA; 2 Department of Cell Biology and Physiology, University of Kansas Medical Center 21638, Kansas City, Kansas, USA; Agroscope, Nyon, Switzerland

**Keywords:** *pombe*, spores, aging

## Abstract

Most fungi can produce dormant, long-lived cells known as spores (e.g., conidia and ascospores). Spores play a critical role in fungal biology and human health, but much about spores is unknown. Here, we investigate factors affecting spore fitness using ascospores of the fission yeast *Schizosaccharomyces pombe* as a model. We found that storage conditions affect spore longevity and that spore health declines over time. We identified a delay in dormancy breaking (germination), decreased asymmetry during cell division, and reduced stress tolerance as aging phenotypes. These results support that *S. pombe* ascospores are affected by both time and experiences during dormancy, highlighting critical features of spore biology and revealing parallels between aging in spores and aging in animal cells.

**IMPORTANCE:**

Fungi are integral to human health and well-being. They also offer scientists easy experimental systems to study facets of life shared with other eukaryotes, including humans. Most studies of fungal cell biology focus on actively growing cells, but fungi can also produce dormant cells known as spores. Spores promote fungal survival and dispersal and are often the agents of infection in pathogenic fungi. In this work, we characterize the spores produced by an easy-to-study model fungus, *Schizosaccharomyces pombe*. We find that the longevity and health of *S. pombe* spores decline over time and in response to heat stress. We characterize several traits associated with stressed and aged spores and identify parallels to aging cells in animals. This study expands the foundation for using *S. pombe* spores as a model system for fungal spore biology and as a model for the aging of non-dividing cells.

## INTRODUCTION

Spores are specialized, dormant cells produced by most fungal species. Some fungi produce spores asexually from mitotically growing cells (conidia), some make spores sexually from the products of meiosis (ascospores and basidospores), and some can make both types of spores (1, 2). While spores are diverse in form and DNA content, both sexual and asexual spores are generally long-lived and tend to show enhanced resistance to stressors, including enzymatic digestion, chemicals, heat, ultraviolet light, and desiccation (1–4). Both types of spores have a specialized, thick spore wall, as well as a dense cytoplasm enriched with protective molecules (e.g., glycerol, mannitol, or trehalose) that give them enhanced stress resistance (3–7). The viscous cytoplasm hinders the movement of molecules within the cell, reducing metabolic rate. Spores can further downregulate their metabolism via modulation of gene expression and, in ascospores, through the polymerization of metabolic enzymes into aggregates (5, 8, 9).

Under permissive conditions, both sexual and asexual spores can undergo germination—the process of dormancy breaking. Germination ultimately results in the loss of the spore’s stress-resistant features and the resumption of growth and cell division, although some stress resistance can persist for a few cell divisions in *Saccharomyces cerevisiae* ascospores (10). The exact stimuli that drive germination vary between fungi, but a carbon source (e.g., glucose) is sometimes required (3, 11–13). Germination of sexual and asexual spores involves isotropic swelling, outgrowth, and cell division, and the process can be influenced by spore age, density, and environmental conditions (e.g., temperature, pH, and nutrients) (3, 4, 14–19).

Spores play critical roles in fungal biology, including facilitating dispersal and allowing fungi to survive environmental stresses (2–4). Spores are extremely common in nature, and many pathogenic fungi, including *Cryptococcus* and *Aspergillus* species, generally infect humans via inhalation of spores (1, 2, 4, 20–22). In addition, plant pathogenic fungi (e.g., the basidiomycete plant pathogen, *Ustilago maydis*) can also spread via spores, resulting in crop losses and food spoilage (4, 23). Understanding spore biology is, therefore, critical to human health and wellness.

More broadly, fungal spores offer an underexplored opportunity to understand dormancy—one of the most widespread cellular survival strategies (24, 25). For example, plants, nematodes, rotifers, and tardigrades all have the potential to experience dormant life stages that contribute to long-term survival under changing, sometimes hostile, conditions (26–29). While humans do not have a systemic dormant state, many human cells are non-proliferative and can even enter a dormant state. These cellular transitions have critical health implications. For example, cancer cells can exhibit a treatment-resistant state of dormancy, where they become quiescent only to resume proliferation after treatment has eliminated primary tumors (25, 30). However, our understanding of the molecular mechanisms of cellular dormancy, in general, is limited.

Ascospores produced by the fission yeast *Schizosaccharomyces pombe* offer a highly tractable model system to explore spore biology and cellular dormancy (31). *S. pombe* generally grows as haploid yeast cells. When *S. pombe* haploids are starved for nutrients, particularly nitrogen, two cells with opposite mating types can mate to form a diploid cell. Under persistent starvation, the diploid cell undergoes meiosis to generate four haploid cells that develop into ascospores, although they are generally just called “spores” in the *pombe* field (3, 32, 33). *S. pombe* researchers have long known that *S. pombe* spores are generally viable for long periods when stored in water at 4°C. Still, the exact limits on the longevity of *S. pombe* spores and the environmental factors affecting it are largely undocumented.

In this work, we use *S. pombe* spores to investigate factors affecting spore health and longevity. We found that spore health is affected by storage temperature, highlighting that experiences during dormancy can impact future health. In addition, *S. pombe* spores display aging phenotypes, making them a tractable model for studying the aging of non-dividing cells. Overall, our results expand our understanding of stress and aging during cellular dormancy.

## RESULTS

### Storage temperature affects spore longevity

We used sexually produced spores from a homothallic (self-mating) strain derived from the commonly used *Schizosaccharomyces pombe* (Lindner isolate [34]). We produced spores by plating haploid cells on malt extract agar (MEA, see Materials and Methods) where the cells mate to form diploids, undergo meiosis, package the four meiotic products into spores, and release the spores from the sacks (asci) that hold them (Fig. S1A). We isolated the spores using a standard protocol that involves treatment with ethanol and glusulase (a snail gut enzyme) to kill vegetative cells (see Materials and Methods [35]). Unless otherwise noted, we stored the spores in water and germinated them on solid, rich media (YEAS, see Materials and Methods) at 32°C.

**Fig 1:**
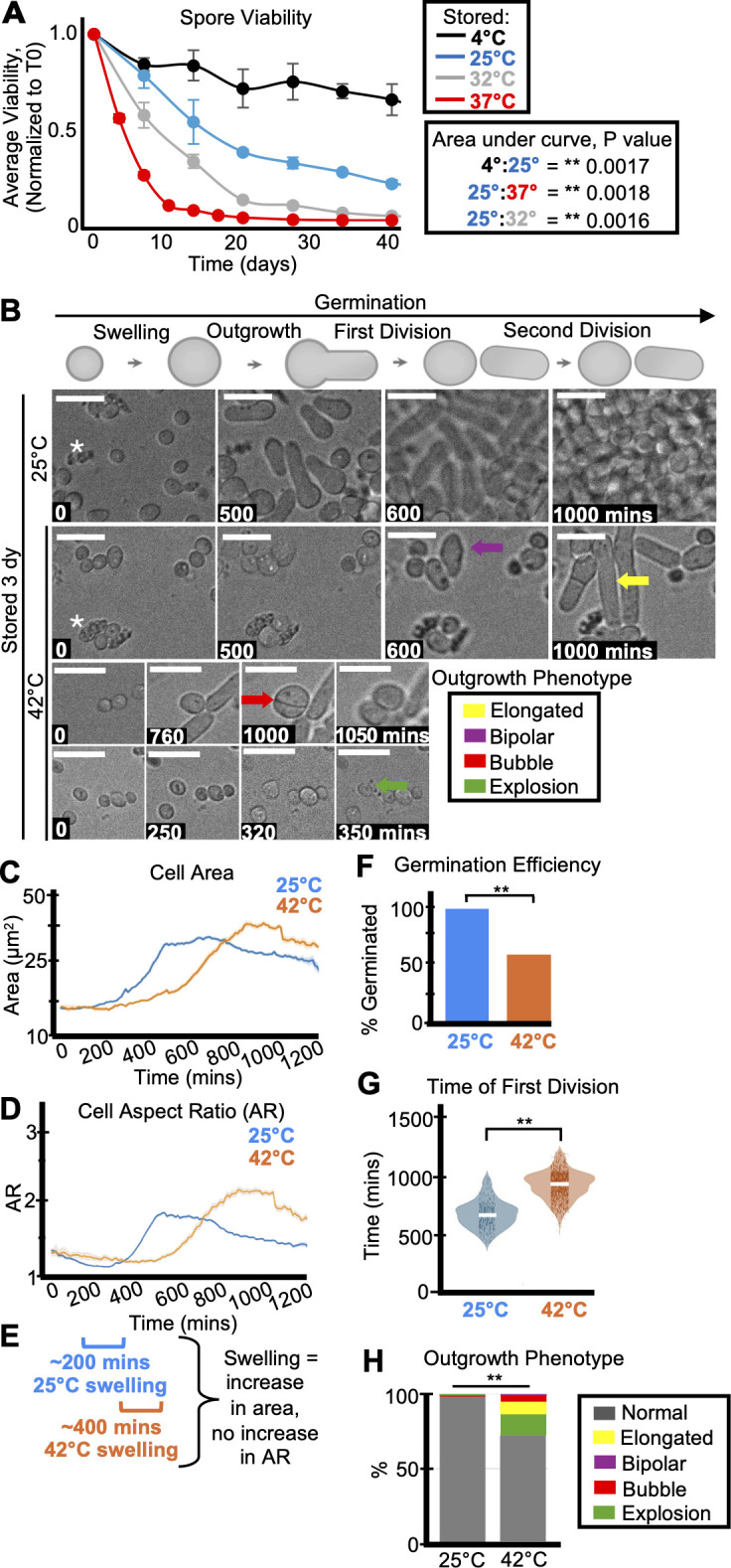
Storage temperature affects spore health and longevity. (**A**) One population of spores in water was split and stored at 4°C, 25°C, 32°C, or 37°C. The stored spores were then sampled over time to assay the number of colony-forming units on rich media (YEAS), which were normalized to the time zero starting point. The average of three replicates is shown, and the error bars represent standard deviations. **P value < 0.01, comparing areas under the curves via *t*-test. (**B**) Time-lapse microscopy images of spores that were stored for 3 days at 25°C (top) or 42°C (bottom) prior to being plated on YEAS to induce germination at 32°C. The spores were imaged every 10 minutes for 1,200 minutes. Scale bars represent 10 µm. The colored arrows represent the following aberrant spore outgrowth phenotypes: elongated outgrowth (yellow), bipolar outgrowth (purple), bubble outgrowth (red), and spore explosion (green). The white asterisks highlight debris. (**C**) Cell area (µm^2^) and (**D**) cell aspect ratio (AR) during germination of spores stored for 3 days at 25°C (blue) or 42°C (orange). The shaded area around each line represents standard error. In some instances, the standard error is thinner than the line. Note that these cell areas provided by the deep learning algorithm are slightly larger than the actual cell areas (see Materials and Methods). (**E**) Swelling is defined as an increase in cell area without an increase in aspect ratio (departure from circularity). The brackets indicate approximate windows of swelling derived from the plots in panels **C** and D. (**F**) The fraction of spores manually observed to germinate by the end of the 1,200-minute time-lapse. *N* > 50 spores per sample. **P-value < 0.01 via Fisher’s exact test. (**G**) Time of first division (time when the AR of a cell exceeded 3) of spores after storage for 3 days at 25°C or 42°C. **P-value < 0.01 via ANOVA test with *post hoc t*-tests for specific comparisons. *N* > 100 spores per sample. (**H**) The percentage of spores displaying the indicated outgrowth phenotypes after storage for 3 days at 25°C or 42°C. *N* > 50 outgrowths per sample. **P-value < 0.01 via Fisher’s exact test.

To understand how spore fitness might change over time, we first assayed spore viability at different storage temperatures: 4°C, 25°C, 32°C, and 37°C. In all conditions, we saw a decrease in spore viability over time, as measured by colony-forming units (CFUs). The viability loss was slowed by storage in colder temperatures (Fig. 1; Fig. S1B through D). For example, spores stored at 37**°**C generally dropped under 50% viability by 10 days, while it took at least 80 days to drop to under 50% viability at 4**°**C (Fig. 1A; Fig. S1B through D). This longevity at 4**°**C in water is less than what has been previously reported for *S. pombe* spores (35% viability after ~1,700 days [3]), perhaps due to differences in strain background, reagents, spore isolation protocols, or spore storage conditions (see Materials and Methods). While not directly comparable due to different conditions, our observations of *S. pombe* spore longevity at 25°C (~20% viability after 60 days) are reasonably similar to previous reports for spores stored at 30°C (~10% viability after 60 days [3]).

In addition, our longevity assays were reproducible across replicates, with some batch effects (Fig. 1A; Fig. S1C; Table S1). Altogether, our results support the conclusion that the temperature experienced during dormancy affects *S. pombe* spore survival. These results are also consistent with previous work showing that some spores from various species lost viability at higher temperatures (1–3, 36, 37). The molecular mechanisms influencing spore heat resistance remain to be fully elucidated; however, environmental conditions (e.g., pH and sugar concentration) during spore formation and storage have been shown to influence both sexual and asexual spore heat tolerance (1, 2).

### Storage temperature affects spore health

To assay how the quality of spores, beyond their viability, might change over time at different storage temperatures, we observed cytological features of germination in time-lapse images. We plated spores on solid, rich media and imaged them every 10 minutes for at least 1,000 minutes (~20 hours) at 32°C (Fig. 1B; Fig. S1E). We classified spores as successfully germinated if they completed one cell division within this timeframe. To facilitate scoring the time-lapses, we trained a deep learning algorithm to identify spores. We then used standard image processing approaches to measure the area and aspect ratio (AR) (ratio of the long to short axis of an ellipse fit to the object) of each tracked cell in each time point. The first division (or successful germination) was defined as the time when a cell’s aspect ratio exceeds 3 (see Materials and Methods). We also scored at least 30 cells per sample manually to confirm the algorithm outputs. We noted that the fraction of spores we observe to germinate is often higher than expected based on our colony-forming unit measurements. This is likely because spores can lose integrity upon death and are not recognized and assayed in the cytological experiments. Consistent with this, we see debris accumulate in the spore storage tubes (as a layer above the spore pellet) as well as in the background of our time-lapses of samples where CFUs have dropped (asterisks in Fig. 1B). In addition, some spores may be capable of completing one cell division but may not be capable of generating a colony. Because of these factors, we consider the colony-forming unit assays as a better measurement of viability, while the cytological analyses reveal additional phenotypes.

At the standard storage temperature of 4°C, we found that fewer visible spores germinated in older samples compared to younger samples, as expected from our CFU analyses (Fig. S2A through D and F through I; Fig. S3A through D). Despite the loss in viability, we found that the viable spores largely remained healthy during storage at 4°C, with no gross defects in germination morphology. In two experiments, we found that older spores (65 and ~400 days) were slower to swell (as defined by an increase in spore area without an increase in aspect ratio) and germinate than 1-day-old samples (Fig. S2A through C and E; Fig. S3A through C and E). We did not, however, observe a difference in swelling or germination timing of spores from a third experiment that compared spores stored for 15 and 397 days at 4°C, suggesting that changes observed at 4°C in germination timing are small and thus within the range of experimental variability of these phenotypes (Fig. S2F through H and J).

We next assayed the health of spores stored at warmer conditions, first comparing 25°C (mild conditions) to 42°C (heat stress) for 3 days. As expected, we found that fewer spores stored at 42°C germinated relative to the spores stored at 25°C (Fig. 1F). The spores stored at 25°C were also faster to swell and divide than spores stored at 42°C (Fig. 1C through E and G; Fig. S4A and B). We observed delayed germination of spores stored at 42°C across multiple time-lapses, new spore isolations, and distinct germination conditions (Fig. S5). These data show that 42°C storage is reproducibly detrimental to spore health. Previous work found that larger *S. pombe* spores germinate faster (38), so we tested if the germination delay in the spores stored at 42°C was associated with decreased size. This was not the case, as the spores stored at 42°C were either not significantly different in size or slightly larger than those stored at 25°C (Fig. S4C and S5G).

We noticed that spores stored for 3 days at 42°C often displayed abnormal outgrowth phenotypes (Fig. 1B, arrows, quantified in Fig. 1H; Fig. S5E). Generally, germinating spore outgrowths are ~4.5 µm prior to the first division, but some spores stored at 42°C produced longer outgrowths (as long as 20 µm), suggesting cell cycle defects (yellow arrows, Fig. 1B [39, 40]). In addition, we observed an increase in polarity defects among the spores stored at 42°C for 3 days (17, 41). Specifically, we observed “bubble”-like divisions, where the cells remain circular and divide in the middle of the “bubble” (red arrow, Fig. 1B). We also observed increased bipolar outgrowths from spores stored at 42°C, where the spore grows from both ends, rather than asymmetrically from one side (purple arrow, Fig. 1B). Finally, we observed an increase in “exploding” spores (green arrow, Fig. 1B) that disintegrate during germination among those stored at 42°C.

We also assayed spores stored at a milder heat stress, 37°C, for 15–28 days. Like the spores stored at 42°C, we saw reductions in germination efficiency of the visible spores, delayed germination, and an increase in abnormal outgrowth phenotypes in the spores stored at 37°C, relative to those stored at 25°C. These effects were observed with spores germinated on rich media at both 32°C and 37°C and on synthetic media at 32°C (Fig. S6 to S8).

To test if storage under heat stress affected spore morphology, we imaged spores using scanning electron microscopy (SEM) and scanning transmission electron microscopy (STEM). We saw no differences in the external appearance of spores stored for 3 days at 25°C or 42°C (Fig. S9A and B). We did see, however, that more of the spores stored at 42°C, relative to those stored at 25°C, had lost the dark-staining appearance of the cytoplasm (Fig. S9C and D). This change is likely due to a change in electron density in the cytoplasm, as areas of the cell that are more electron dense allow fewer transmitted electrons, and thus appear darker; conversely, thinner areas permit more transmission and appear lighter (42, 43). Altogether, these observations demonstrate that storage in heat-stressed conditions negatively impacts spore health.

### Storage medium affects spore longevity

**Fig 2:**
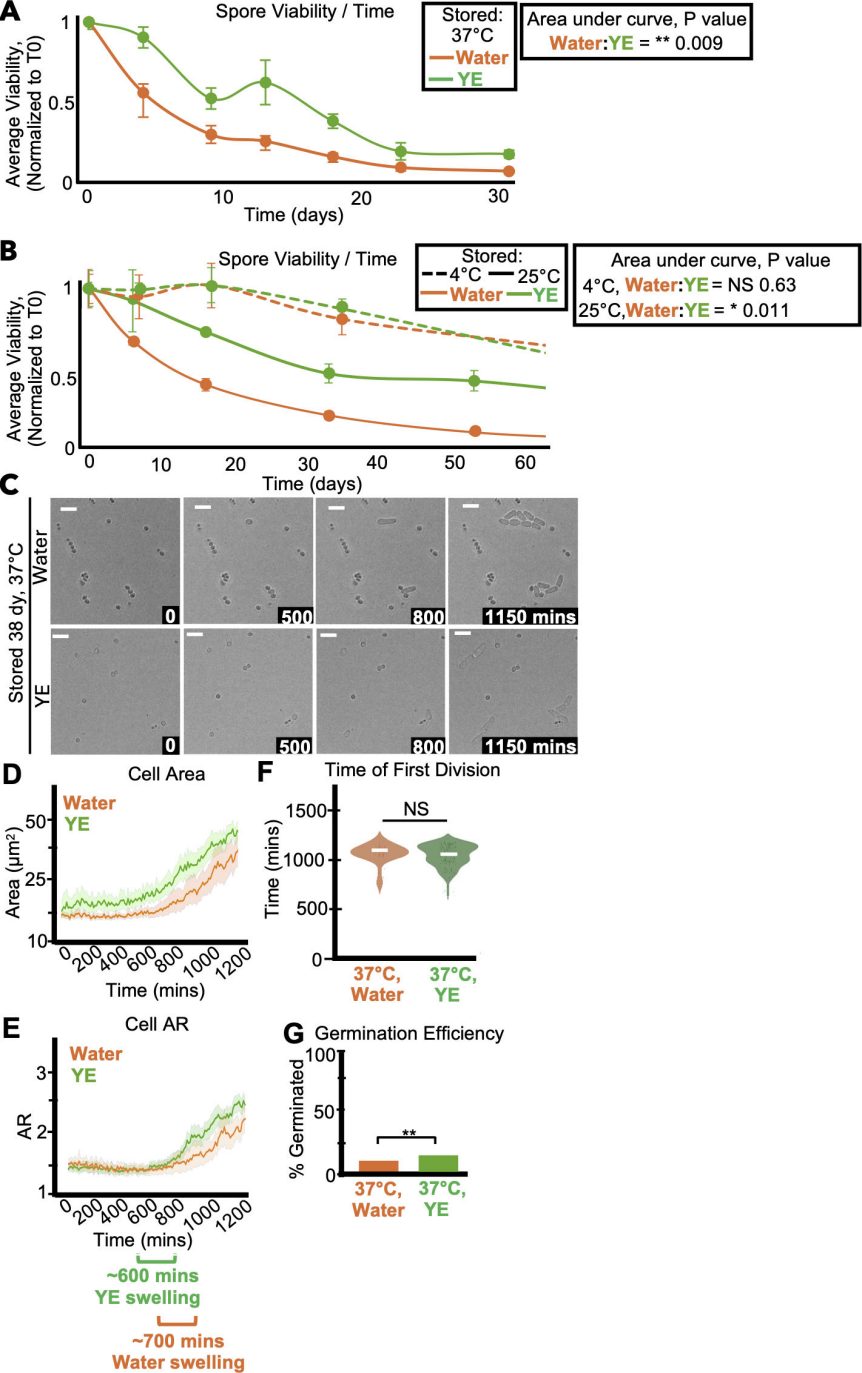
Storage media affects spore longevity. (**A**) One population of spores was split and resuspended in either 0.5% yeast extract solution without glucose or water and then stored at 37°C. The same experiment was performed in panel **B**, but the spores were stored at 4°C or 25°C. In both experiments, the stored spores were then sampled over time to assay the number of colony-forming units on rich media (YEAS), which were normalized to time zero starting point. We show the average of three replicates, and error bars represent the standard deviation. **P value < 0.01, *P value < 0.05, comparing areas under the curves via *t*-test. (**C–G**) Imaging analyses of spores stored for 38 days at 37°C in either water or yeast extract solution before being plated on rich media (YEAS) to induce germination at 32°C. Cells were imaged every 10 minutes for 1,200 minutes. (**C**) Time-lapse microscopy images of germinating spores. The time in minutes is displayed, and the scale bars represent 10 µm. (**D**) Cell area (µm^2^) and (**E**) cell aspect ratio during germination. The shaded area around each line represents standard error. In some instances, the standard error is thinner than the line. Note that these cell areas provided by the deep learning algorithm are slightly larger than the actual cell areas (see Materials and Methods). (**F**) Time of first division (time when the AR of a cell exceeded 3). **P-value < 0.01 via ANOVA test with *post hoc t*-tests for specific comparisons. *N* > 100 spores per sample. (**G**) The fraction of spores manually observed to germinate by the end of the 1,200-minute time-lapse. *N* > 50 spores per sample. **P-value < 0.01 via Fisher’s exact test.

Ascospores isolated from *Saccharomyces cerevisiae* and conidia from *Aspergillus* can incorporate labeled nucleotides and amino acids, suggesting spores may carry out some transcription and translation (44, 45). We, therefore, wanted to test if a nutrient-dense storage medium affects *S. pombe* spore health by comparing spores stored in water to those stored in a solution of 0.5% yeast extract (YE). We stored the spores at 4°C, 25°C, and 37°C and measured spore viability over time by assaying colony-forming units. Spores stored in YE at 25°C and 37°C generally survived longer than matched samples stored in water (Fig. 2A and B). However, at 4°C, we did not observe repeatable differences between spores stored in YE compared to those in water (Fig. S10A and B). Importantly, we did not identify which features of YE support spore longevity better than water at warmer temperatures. We speculate it is the presence of nutrients, but we did not rule out any potential contributions of osmolarity or slight pH differences.

We also assayed the effects of storage media on spore health by cytologically comparing cells stored at 4°C, 37°C, and 42°C in either YE or water. We observed a trend of more and faster germination among spores stored in YE relative to those stored in water. However, these effects (except germination efficiency after 38 days at 37°C) were not statistically significant in individual experiments (Fig. 2C through F; Fig. S10 to S12). Together, our results suggest that storage in YE significantly affects spore survival at warmer temperatures but does not significantly influence the health of the viable spore population.

### Age affects spore health

**Fig 3:**
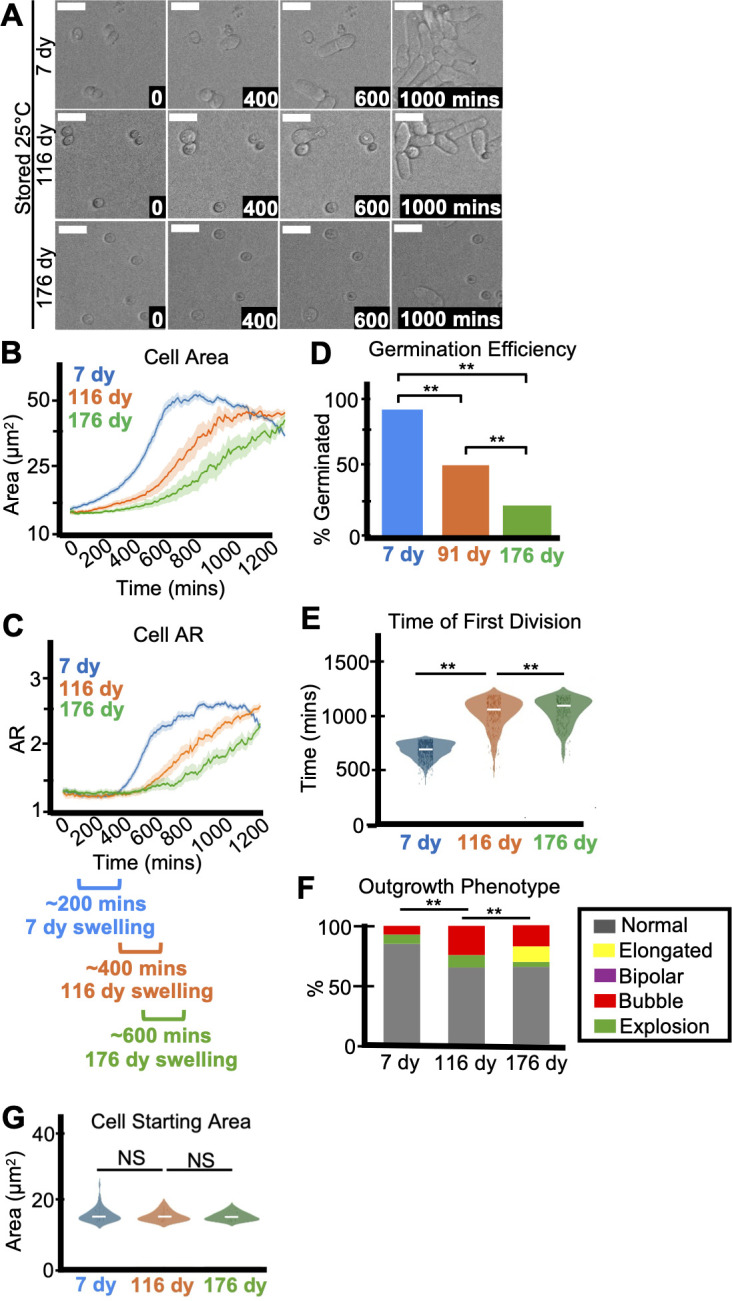
Age affects spore health. Imaging analysis of spores stored at 25°C for 7, 116, or 176 days in water before being plated on rich media (YEAS) to induce germination at 32°C. Cells were imaged every 10 minutes for 1,200 minutes. (**A**) Time-lapse microscopy images of germinating spores. The time in minutes is displayed, and scale bars represent 10 µm. (**B**) Area (µm^2^) and (**C**) aspect ratio during germination. The shaded area around each line represents standard error. In some instances, the standard error is thinner than the line. Note that these cell areas provided by the deep learning algorithm are slightly larger than the actual cell areas (see Materials and Methods). (**D**) The fraction of spores manually observed to germinate by the end of the 1,200-minute time-lapse. *N* > 50 spores per sample. **P-value < 0.01 via Fisher’s exact test. (**E**) Time of first division (time when the AR of a cell exceeded 3). **P-value < 0.01 via ANOVA test with *post hoc t*-tests for specific comparisons. *N* > 100 spores per sample. (**F**) The percentage of spores displaying the indicated outgrowth phenotypes. *N* > 50 outgrowths per sample. **P-value < 0.01 via Fisher’s exact test. (**G**) Starting area (µm^2^) of spores. *N* > 50 spores per sample. NS indicates not significant via ANOVA test with *post hoc t*-tests for specific comparisons.

Our data (Fig. 1A) demonstrate that spores lose viability over time, and our cytological observations of spores stored at 4°C suggest that spore health can decline over time, but the effects were small and varied between experiments (Fig. S2 and S3). We predicted that any age-associated effects would manifest more quickly and be more obvious with spores stored at warmer temperatures. We, therefore, compared germination phenotypes of spores stored at 25°C for 7, 116, or 176 days. As expected, significantly fewer of the visible 116- and 176-day-old spores germinated than the 7-day-old spores (Fig. 3A and D). The aged spores were also significantly slower to swell, outgrow, and divide than the 7-day-old spores (Fig. 3B, C, and E). The differences in germination time were repeatable across distinct spore isolations (Fig. S13), not due to size differences between spores (Fig. 3G) or spore concentration (Fig. S14), and were observed across different germination conditions (Fig. S15 and S16). We also noted significantly more abnormal germination morphologies in the aged spores (Fig. 3F; Fig. S13F), like those observed in the young spores stored for 3 days at 42°C (Fig. 1H; Fig. S5E).

Finally, similar to heat-stressed spores, we observed significantly more spores with light-staining (i.e., less electron-dense) cytoplasm in older spores compared to young (Fig. S9C and D). We also saw more deflated, shrunken spores in the older samples than the younger samples (Fig. S9A and B). Overall, these observations demonstrate that age can negatively impact spore health. The mechanisms leading to this age-related decline could be due to classical hallmarks of aging (e.g., genomic instability, epigenetic alterations, loss of proteostasis, mitochondrial dysfunction, etc. [43]), but additional experiments are required to test these hypotheses.

### Age and stress affect the asymmetry of spore germination

**Fig 4:**
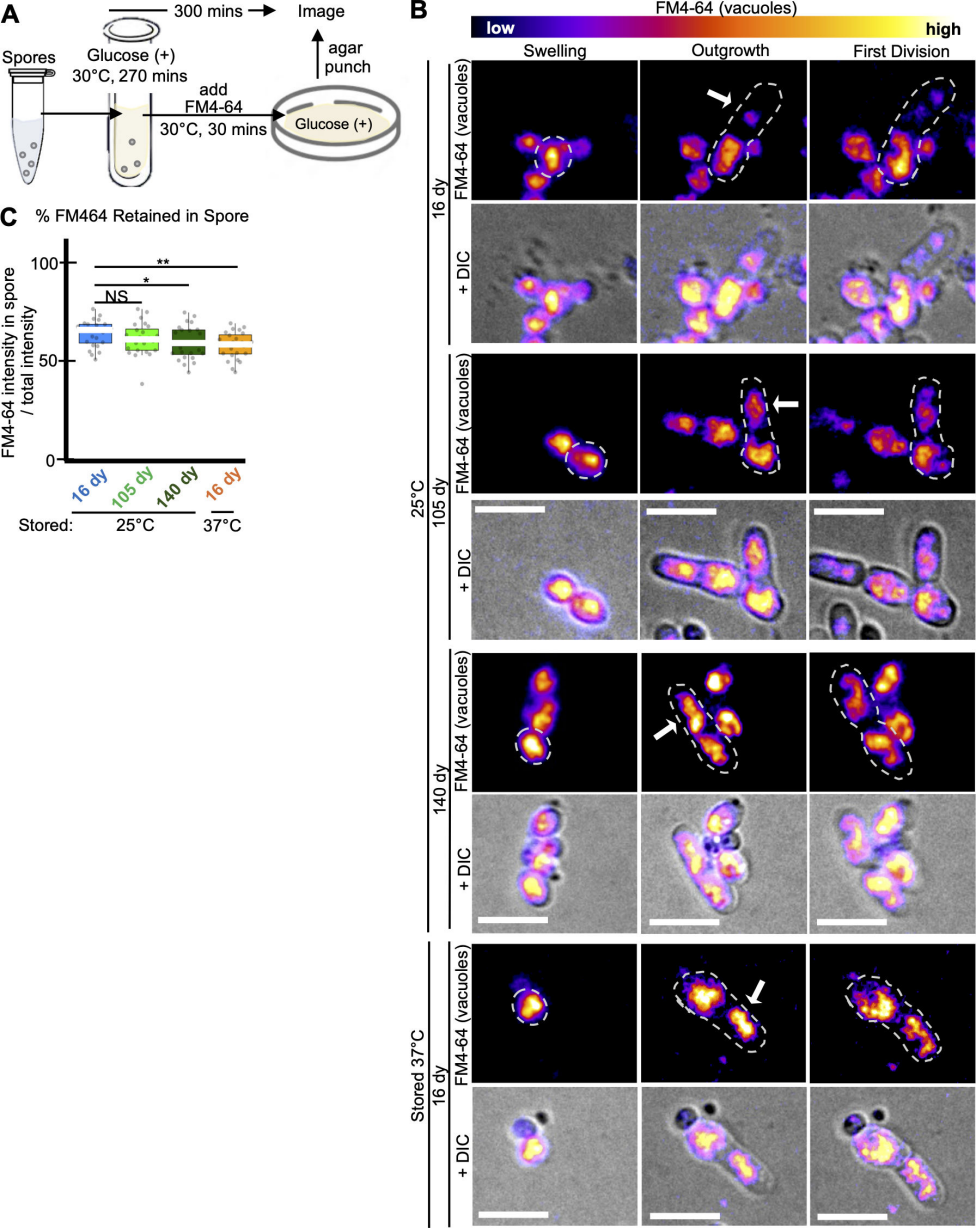
Age affects asymmetry of vacuole segregation during germination. Imaging analyses of vacuole segregation during germination of spores previously stored for 16, 105, or 140 days at 25°C in water or for 16 days at 37°C in water. (**A**) Cartoon describing experimental approach. Spores were resuspended in rich liquid media (YES) at 30°C for 270 minutes to induce germination. The vacuole dye FM4-64 was then added, and the cells were grown for an additional 30 minutes before being plated on solid rich media (YEAS). The cells were then imaged on the agar at 32°C using time-lapse microscopy. (**B**) Time-lapse microscopy images of FM4-64-stained spores for 20 hours. The brightness and contrast are not the same for all images but were adjusted so the spore bodies appeared to have similar levels of signal. Scale bars represent 10 µm. (**C**) Percentage of the total FM4-64 signal retained in the spore body versus the germ tube outgrowth. *N* > 30 spores. **P-value < 0.01, *P-value < 0.05, and NS, not significant, via Fisher’s exact test. Error bars show standard error.

Previous work demonstrated intracellular asymmetry in germinating *S. pombe* spores, showing that vacuoles are preferentially retained in the round end of germinating spores, as opposed to the outgrowth (19). We wondered if the decreased asymmetry we observed in the shape of germinating heat-stressed (Fig. 1B and H; Fig. S5E, S6E, S7E, and S8E) and aged spores (Fig. 3F; Fig. S13F) extended inside the cells to the vacuole. To test this, we assayed the partitioning of dye-stained (FM4-64) vacuoles during germination of spores stored at 25**°**C for 16, 105, and 140 days, as well as 37**°**C for 16 days (Fig. 4A). We saw that the 25**°**C-stored, 16-day-old and 105-day-old spores retained most (64% and 60%, respectively) of the vacuole signal in the spore, with less FM4-64 signal in the outgrowth (Fig. 4B and C). However, the 25**°**C-stored, 140-day-old spores and the 37**°**C-stored, 16-day-old spores had significantly more vacuole straining entering the outgrowth (Fig. 4B and C). This phenotype of decreased vacuole asymmetry at the first division in older and heat-stressed spores persisted between spore preparations (Fig. S17 and S18), indicating that spores’ ability to asymmetrically segregate vacuoles can decline due to heat stress and due to aging.

### Prior stress and aging affect spore stress tolerance

**Fig 5:**
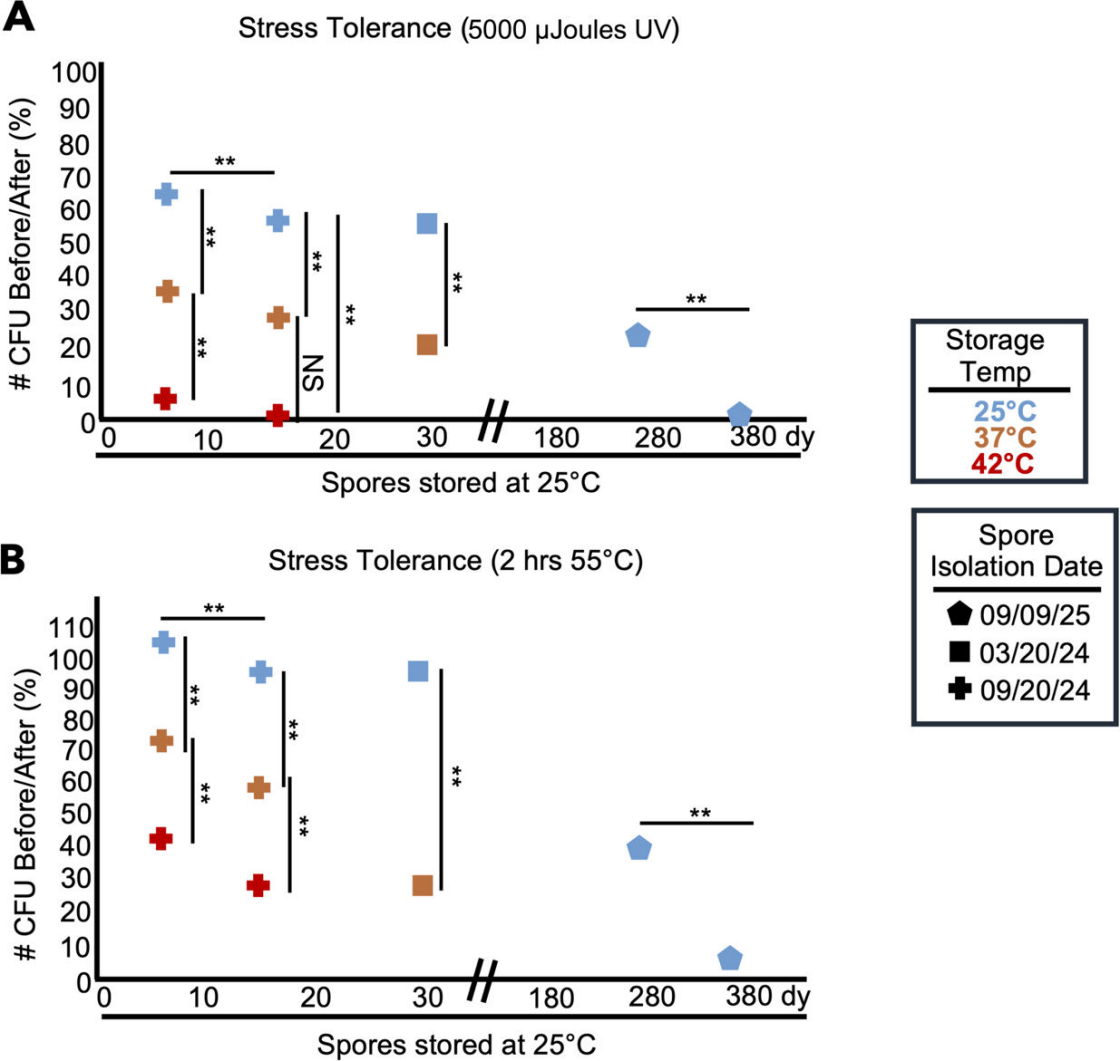
Age and stress affect spore stress tolerance. The colony-forming units of spore samples previously stored at 25°C (blue) for 7, 15, 30, 261, and 375 days, 37°C (orange) for 7, 15, and 30 days, or 42°C (red) for 7 and 15 days were determined by plating on rich media (YEAS) at 32°C both before and after an acute stress. (**A**) The ratio of surviving CFUs before and after exposure to 5,000 μJ UV or (**B**) a 1-hour heat shock of 55°C. Symbols represent different spore isolations. Each symbol represents the average of five replicates. We compared individual spore isolations over time or individual temperatures within a spore isolation, but we never compared two separate spore isolations over time. We used a *t*-test (when comparing two samples) or an ANOVA test with *post hoc t*-tests (when comparing three samples). **P-value < 0.01. NS, not significant.

A key spore phenotype is that they display resistance to stresses (1–3, 46–50). We hypothesized that past experiences and age could affect stress resistance in spores. Such an effect has been observed in both sexual and asexual spores*,* where heat resistance can either positively or negatively correlate with age, depending on species (1, 2, 51). To test our hypothesis in *S. pombe*, we counted the number of colony-forming units produced by spore populations before and after heat stress (55°C) and before and after exposure to ultra-violet (UV) light (see Materials and Methods). We found that a significantly larger fraction of the spores stored at 25°C survived both heat and UV shock compared to age-matched spores stored at 37°C or 42°C (Fig. 5). Similarly, we found that young spores had higher resistance to heat and UV stress than older spores (Fig. 5). These experiments demonstrate that age and storage conditions negatively influence *S. pombe* spore’s ability to tolerate stress and highlight that effects can vary widely between spores of different species.

## DISCUSSION

Understanding spore formation, maintenance, and germination is crucial for understanding the biology of fungi. But much remains to be learned about these questions, even in intensely studied and tractable model systems like *S. pombe* (31). Here, we aimed to help fill that gap by investigating influences on the health and longevity of *S. pombe* spores. We established parameters for *S. pombe* spore longevity under varying conditions (Fig. 1A). Similar to work in various species, we found that storage temperature significantly impacts spore health and longevity (1–3, 46–55).

To reduce the labor associated with studying germination, we improved our previously developed artificial intelligence-assisted image scoring approaches to automate the scoring of the key germination phenotypes (swelling and first division timing [56]). In addition, we identified several phenotypes associated with spore stress and aging: decreased germination efficiency, delayed germination, and altered spore outgrowths.

Consistent with standard practices within the *S. pombe* field, we found that some spores could maintain viability for long periods when stored in water at 4°C. We were surprised to find, however, that a sizeable fraction of spores stored at 4°C died within 100 days of storage under our laboratory conditions. Still, the spores that remained viable at 4°C appeared grossly normal and showed mild or no obvious health defects. Spores stored at higher temperatures, however, exhibited accelerated loss of health and viability.

For future analyses, we find it interesting that storage in yeast extract can increase spore longevity. This is consistent with previous work that showed that the sugar and organic acid composition of the storage media can influence ascospore heat resistance (1, 2). This suggests that spores might utilize their nutrient environment, as has been suggested in *Saccharomyces* ascospores and *Aspergillus* conidia (44, 45). However, it could also be due to another aspect of the media, such as pH or osmolarity. Additional experimental investigation is required to distinguish between these possibilities.

We are also particularly interested in the causes and consequences of the breakdown in vacuole retention in aged spores. We speculate that the asymmetry of the first division after germination may promote rejuvenation of the lineage founded by the spores. This idea is based on work in *Saccharomyces cerevisiae* (budding yeast), which has a similar asymmetric division style as the typical first and second divisions of *S. pombe* spores. For example, in vegetatively growing budding yeast, debris (e.g., misfolded proteins/RNAs, extrachromosomal circles, and aggregates) is retained in the mother cell during cell division. This asymmetric cytoplasmic inheritance promotes the rejuvenation of the daughter cell (the bud), and this process is less efficient in aged mothers (57–59). *S. pombe* spores could similarly use asymmetric division to purge debris in the first daughter cell. If so, this process is breaking down over storage time in spores.

Further parallels can be drawn between spores and stationary phase cells. Both budding and fission yeast can enter a temporary and reversible stationary phase known as quiescence that shares evolutionary conservation across eukaryotes, including multicellular animals (60, 61). Quiescence involves a large-scale remodeling of cellular processes that share characteristics with spores, such as a reduced metabolic state and an altered cell wall. Quiescent cells also display classic hallmarks of aging, such as DNA mutations and epigenetic alterations (61, 62). Therefore, understanding spore biology could provide insights not only into the molecular mechanisms controlling quiescence but also how aging phenotypes manifest in non-dividing cell types.

Parallels between spore germination and animal stem cells are also notable. First, the asymmetric spore germination phenotype is reminiscent of the asymmetric divisions of human stem cells, where one daughter cell retains stem cell properties and the other differentiates into a specialized cell. Interestingly, asymmetric inheritance of lysosomes (the equivalent of fungal vacuoles) is an important aspect of this differentiation. However, older stem cells tend to lose their ability to divide asymmetrically and produce two daughter cells with similar characteristics, causing tissue decline associated with aging (63, 64). In addition, we found that aged spores are slower to break dormancy than younger spores. This also mirrors an aging phenotype in human stem cells, where aged, non-dividing stem cells become more resistant to activation, generating a progressive age-associated delay in progressing through the cell cycle (65, 66). Alterations to the genome, the epigenome, and the proteome underlie the molecular mechanisms leading to the age-related decline in stem cells, and these changes greatly resemble hallmarks of aging in dividing cells (67). These similarities between spores and stem cells suggest that discoveries about experimentally tractable fungal spores may sometimes reflect deeply conserved biology and thus be broadly relevant.

We aspire to have this study provide parameters and phenotypes that will be helpful in future studies addressing the molecular mechanisms underlying spore longevity and stress resistance phenotypes in the highly tractable *S. pombe* system. We anticipate that at least some of these mechanisms may be shared with other fungal sexual and asexual spores (e.g., pathogens [4]) and other non-dividing eukaryotic cell types (e.g., mammalian quiescence, oocytes, and diapause [61, 68, 69]).

## MATERIALS AND METHODS

### Inducing sporulation

We cultured SZY643 (*h90, leu1-32,* and *ura4-D18* [70]) in 5 mL of YES rich media (0.5% yeast extract, 3% glucose, 250 mg/L of adenine, lysine, histidine, leucine, and uracil) overnight at 32°C with shaking. We then diluted the culture to 10^−3^, 10^−4^, and 10^−5^. We plated 100 µL of the 10^−3^ dilution on five separate malt extract agar plates (2% Difco Bacto Agar, 30 g/L Bacto-malt extract, 5 mg/L adenine, histidine, leucine, and uracil, pH adjusted to 5.5 with NaOH) to induce mating and sporulation. We also plated 100 µL of the 10^−4^ and 10^−5^ dilutions on rich media, YEAS (same as YES above with 2% agar). We counted the colony-forming units on the YEAS plates after 3–5 days to estimate the number of cells plated on the MEA. We incubated the MEA plates at 25°C for 9 days. By day 9, the cells (i) self-mated, (ii) underwent meiosis to produce spores, and (iii) naturally broken down the spore sac to release the spores (Fig. 1A). Using a cell scraper (Fisher Scientific: 353086) and water, we harvested the total mass of all spots off the five MEA plates into a 50 mL conical tube with 20 mL of water and continued with spore isolation methods (below).

### Isolation of spores

We killed vegetative cells to isolate spores using glusulase, followed by ethanol (35). We added 100 µL of glusulase (Sigma-Aldrich: G0876) to 20 mL of spores in water and placed the tubes at 32°C overnight with shaking. The next day, we added 20 mL of 60% ethanol for 10 minutes at room temperature. We pelleted the spores by centrifugation, washed them twice with 25 mL of water, and resuspended them in 50 mL of water. We then stored the spores in 2 mL aliquots in water (unless otherwise noted) at various temperatures in screw cap tubes in a light-protected box. To limit phenotypes introduced by repeated vortexing and to prevent contamination, each aliquot was sampled once or for one day of experiments.

### Longevity assays

We plated dilutions of spores onto YEAS and counted the colonies after 3 and 5 days of growth at 32°C (unless otherwise noted). To calculate the area under the curves, we used Excel. There is not a single function in Excel that can calculate the area under the curve; therefore, we used the trapezoidal rule. To do so, we divided the area under the curve into trapezoids and calculated the area of each trapezoid using the formula Area = (*a* + *b*)/2 × *h*, where *a* is the base length of one side, *b* is the base length of the other side, and *h* is the height. We calculated the trapezoidal area for each *x*-axis interval and then summed the areas together to get the total area under the curve.

### Vacuole, FM4-64 staining

We found that dormant spores did not efficiently take up the dye FM4-64 (Invitrogen: T13320), but the cells became permeable to the dye during germination. Therefore, we placed the spores in YES liquid media for 270 minutes at 30°C with shaking to induce germination prior to adding FM4-64 (Invitrogen: T13320) to the YES media at a concentration of 10 µM. We then incubated the cells for an additional 30 minutes with shaking at 30°C to allow staining (Fig. 5A). We then washed the germinating spores twice with water and subjected them to time-lapse microscopy of germination.

### Time-lapse microscopy

The samples used in each experiment are described in Table S2. Some spore preparations were used in multiple experiments; for example, a distinct aliquot from a spore preparation used in one experiment as a young sample could later be used in a subsequent experiment as an aged sample. We diluted the spores to 10^−3^ in water and then plated 100 µL of the 10^−3^ dilution onto germination media (media containing glucose, usually YEAS, with the exception of Fig. S3, S5, S7, and S15, which utilize PMG [3 g/L potassium hydrogen phthalate, 2.2 g/L Na_2_HPO_4_, 3.75 g/L glutamic acid, monosodium salt {Sigma G-5889}, 20 g/L glucose, 20 mL/L salts {0.26 M MgCl_2_·6H_2_O; 4.99 mM CaCl_2_·2H_2_O, 0.67 M KCl, and 14.1 mM Na_2_SO_4_}, 1 mL/L vitamins {1,000× stock: 2.20 mM pantothenic acid, 81.2 mM nicotinic acid, 55.5 mM inositol, and 40.8 µM biotin}, 0.1 mL/L minerals {10,000× stock: 80.9 mM boric acid, 23.7 mM MnSO_4_, 13.9 mM ZnSO_4_·7H_2_O, 7.40 mM FeCl_2_·6H_2_O, 2.47 mM molybdic acid, 6.02 mM KI, 1.60 mM CuSO_4_·5H_2_O, and 47.6 mM citric acid}, and 2% agar]). We then took a 0.6-cm-diameter punch of the agar and placed it upside down into an 8-well uncoated dish (Ibidi: 80826), trapping the cells between the agar and the dish for imaging. We then imaged the cells on a Ti2 widefield microscope (Nikon) through a 60× Plan Apo (NA 1.4, Nikon) oil objective. We imaged every 10 minutes for at least 1,000 minutes (~20 hours), collecting transmitted light using the Prime 95B camera (Photometrics), using perfect focus on a single Z-slice. For the FM4-64 time-lapse, we collected the FM4-64 signal (and transmitted light) every 15 minutes for 1,000 minutes (~20 hours) on the same microscope and objective using an mCherry optical configuration. The excitation was centered at 586 nm, and the emission was collected through an EM610/75 filter, again using perfect focus on a single Z-slice.

### Deep learning algorithm

We implemented a custom-trained Cellpose (version 3 [71]) model for spore tracking and measurements (area and aspect ratio). To create a training set, we labeled all cells in 35 random transmitted light images of spores to make image-mask pairs. Using the training set and starting from the pretrained cyto2 model, the Cellpose training module ran for 1,000 epochs, with an initial learning rate of 0.005 and a weight decay of 0.00001. We saved the model after training and used it for inference in further image analysis. The training and analysis scripts were custom written in Python, and the training computation was performed on an Ubuntu Linux 18.04 workstation with an NVIDIA RTX A6000 GPU. The scripts we generated are all publicly available in the Stowers Original Data Repository (ODR) at https://www.stowers.org/research/publications/LIBPB-2559.

### Plot generation

We used the area and AR measurements calculated by the algorithm for all data except the area of germinated spores, which was measured manually (described below). If a time-lapse temporarily lost focus, the data at that time point appear as a sharp dip or peak in the data (e.g., Fig. S3, 438-day-old sample at 650 minutes). If a time-lapse lost focus for more than five time points, we excluded the data from analysis. To plot time to germination, we scored only unique particles with an AR less than 1.4 in time point zero (these are circular and therefore likely spores). We then designated the time when that object reached an AR greater than 3.0 as the time of the first division. This cutoff was determined by manually comparing AR and division status of >50 cells, which tend to divide at a predictable size and had an average aspect ratio of 3.1 at division.

To plot germination efficiency, we scored using data collected manually by eye. We scored at least 100 spores per sample and counted how many successfully underwent first division before the end of the time-lapse. We then divided that number by the total spores scored. We also scored the outgrowth phenotype manually by eye. We counted at least 50 outgrowths per sample and determined if it was normal, elongated (more than 6 µm), bubbled, exploded, or bipolar. If a spore was both bipolar and elongated, we counted it as bipolar.

To plot the starting area for all spores, we used data collected by the deep learning algorithm. We calculated the area (µm^2^) of unique particles with an AR less than 1.4 at time point zero. Note that the cell areas provided by the deep learning algorithm (e.g., starting spores ~10–15 µm^2^) are larger than the actual cell areas (e.g., starting spores ~7–9 µm^2^) due to the automatically generated cell masks extending slightly beyond the cell boundaries. To determine the starting area for germinated spores, we scored using data collected manually. We calculated the area (µm^2^) of spores at time zero that went on to successfully undergo the first cell division. The scripts we generated are all publicly available in Stowers ODR at https://www.stowers.org/research/publications/LIBPB-2559.

### Electron microscopy

We stored the spores at 25°C or 42°C for varying amounts of time and then completed either STEM or SEM. For STEM, we fixed the spores in 1.5% KMnO_4_ on ice for 30 minutes, rinsed with ultrapure water five times for 10 minutes each, and embedded the spores in 1% low melting agarose (72). We cut the agarose block into pieces less than 1 mm^3^, prior to serial ethanol dehydration. The dehydration steps were performed for 15 minutes at room temperature for 30%, 50%, 70%, 80%, 90%, and three 100% incubations. We then moved the samples into Hard Plus resin (Electron Microscopy Sciences) with an accelerator, starting with a 25% incubation for 2.5 hours, then into a 50% incubation overnight at room temperature, 75% the next day, then 100% and microwaved (Pelco) at 250 W for 3 minutes and incubated overnight. The next day, we did three changes of 100% resin, each microwaved at the same settings before embedding and hardening at 60°C for 48 hours. We cut sections at 80 nm using a Leica Actos ultramicrotome and post-stained the grids for 3 minutes with 1% Sato’s Triple Lead, 4 minutes with 4% uranyl acetate in 70% methanol, and 5 minutes with 1% Sato’s Triple Lead. We imaged the grids on a Zeiss Merlin SEM with STEM detector at 30 kV and 700 pA.

For SEM, we fixed the spores in a 50 mM fixative containing 2.5% glutaraldehyde, 2% paraformaldehyde, 1 mM CaCl_2_, and 1% sucrose buffered with 50 mM Na Cacodylate at pH 7.4 at 25°C for 2 hours and stored them in fixative overnight at 4°C. We then processed the spores using a TOTO protocol (73). Briefly, we rinsed the spores five times for 5 minutes each in ultrapure water, incubated in 1% tannic acid for 1 hour at room temperature on a nutator, washed with the same regime as above, incubated in 1% aqueous osmium tetraoxide for 1 hour at room temperature on a nutator, washed again as above, incubated in 2% filtered aqueous thiocarbohydrizide for an hour at room temperature on a nutator, washed again, followed by another hour in 1% osmium tetraoxide for an hour at room temperature. After a final rinse, we dehydrated the spores in ethanol for 10 minutes through 30%, 50%, 70%, 90%, and three 100% washes and critical point dried with a Tousimis Samdri-795. We then mounted a section of Kimwipe on a stub with carbon tape and sputter coated (Leica ACE 6000) with 4 nm of Au/Pd. The spores were then sprinkled onto the stub, which was further coated with 7 nm of carbon to mitigate spore charging with little to no additional surface texture. We then imaged the spores at 3 kV and 50 pA with an In Lens SE2 detector on a Zeiss Merlin SEM and processed the images using Fiji (74) and the CLAHE function (75) with the following parameters: 127, 256, and 1.5.

### Stress tolerance experiments

We stored the spores at 25°C, 37°C, or 42°C for varying amounts of time and then completed the following stress experiments. For heat stress, we plated dilutions of spores on YEAS plates before heat stress and after incubation at 55°C for 1 hour. We then grew the plates at 32°C for 3 days and counted the number of CFUs. We repeated the counting at 5 days to include any slow-growing colonies. We calculated the percent survival by dividing the number of CFUs on the heat-shocked plate by the number of CFUs on the untreated control plate and averaged the results from three replicates for each sample. For UV stress, we plated dilutions of spores on two YEAS plates. One of the plates was not subjected to UV (control), but the other was exposed to 5,000 μJ of UV light (254 nm). We then incubated the plates at 32°C for 3 days, counted the number of CFUs, and then counted them again at 5 days. We calculated the percent survival by dividing the number of CFUs on the UV-treated plate by the number of CFUs on the untreated control plate.

## Data Availability

All the reagents used in this study are available upon request. Original data underlying this manuscript can be accessed from the Stowers Original Data Repository at https://www.stowers.org/research/publications/LIBPB-2559. This includes all raw images, as well as the Deep Learning scripts used for image analysis and plotting.

## SUPPLEMENTAL MATERIAL

Supplemental figures spectrum.01710-25-s0001.pdf Figures S1 to S18.

Table S1 spectrum.01710-25-s0002.xlsx Raw data for experiments.

Table S2 spectrum.01710-25-s0003.xlsx Summary of samples used in the experiments.

ASM does not own the copyrights to Supplemental Material that may be linked to, or accessed through, an article. The authors have granted ASM a non-exclusive, world-wide license to publish the Supplemental Material files. Please contact the corresponding author directly for reuse.
