# Supplementary material for: Impacts of stress and aging on spore health in Schizosaccharomyces pombe
Source: Microbiol Spectr. 2025 Nov 7;13(12):e01710-25. doi: 10.1128/spectrum.01710-25 (PMC12671135; doi:10.1128/spectrum.01710-25)
Supplement: Supplemental figures — Figures S1 to S18. [file spectrum.01710-25-s0001.pdf]

Figure S1. Storage temperature affects spore health and longevity. (A) Cartoon of the spore isolation approach used for all experiments (see methods for more details). A haploid strain proficient in mating-type switching (h90) was cultured in rich media (YES) then plated on sporulation media (MEA) at 25°C for 9 days. The spores were then scraped into tubes and the remaining vegetative cells were killed with glusulase and ethanol treatment. Pictures of the MEA plates after 3 and 9 days at 25°C are shown on the right, highlighting cells that are undergoing 1. Mating, 2. Meiosis, and 3. Spore release. Scale bars represent 10 µm. (B) One population of spores stored in water was split and stored at 4°C, 25°C, 32°C, or 37°C. The stored spores were then sampled over time to assay the number of colony-forming units (CFUs) on rich media (YEAS), which were normalized to the time zero starting point. The average of 3 replicates is shown and the error bars represent standard deviations. ** indicates p value < 0.01, comparing areas under the curves via t-test. These are the same data as shown in Figure 1A with more timepoints displayed. (C) This plot follows the same format as (B) but shows the variability present within and between experiments. Batch 1 (orange shades) is a distinct experiment from that shown in (B) and each of three replicates of spores stored at 4°C (dotted lines) and 25°C (solid lines) is shown. Batch 2 (blue shades) is the same data as shown in (B) for spores stored at 4°C (dotted lines) and 25°C (solid lines), except each individual replicate is plotted. (D) An independent, but analogous experiment as that presented in (B) and (C) where the viability of spores stored at 25°C, 37°C, 42°C, and 55°C was assayed over time in two batches. (E) To prepare cells for imaging via timelapse microscopy experiments in this study, we placed spores on agarose plates (YEAS or PMG). We then took a punch of this agar plate and transferred the agar plug to an 8-well Ibidi dish, placing it upside down, trapping the spores between the plug and dish.

Figure S2. Storage temperature affects spore health. (A-E) Imaging analyses of spores stored at 4°C for one day or 405 days before being plated on rich media (YEAS) to induce

germination at 32°C. Cells were imaged every 10 minutes for 1000 minutes. (A) Timelapse microscopy images of germinating spores. The time in minutes is displayed and the scale bars represent 10 µm. (B) Cell area (µm^2) and (C) cell aspect ratio (AR) during germination. The shaded area around each line represents standard error. In some instances, the standard error is thinner than the line. Note that these cell areas provided by the deep learning algorithm are slightly larger than the actual cell areas (see methods). (D) The fraction of spores manually observed to germinate by the end of the 1,000-minute timelapse. N>50 spores per sample. ** indicates p-value < 0.01 via Fisher's exact test. (E) Time of first division (when the AR of a cell exceeded 3). ** indicates p-value <0.01 via ANOVA test with post-hoc t-tests for specific comparisons. N>100 spores per sample. The experiments in (F-J) duplicate those shown in (A-E), but the spores were stored for 15 days or 397 days.

Figure S3. Storage temperature affects spore health. Imaging analyses of spores stored at 4°C for one, 65, or 438 days before being plated on media to induce germination at 32°C. Cells were imaged every 10 minutes for 1,400 minutes. (A) Timelapse microscopy images of spores germinating on rich media (YEAS, left) and synthetic media (PMG, right). The time in minutes is displayed and the scale bars represent 10 µm. The 438-day-old sample on YEAS lost focus at the 650-minute time frame (see methods for details on how such samples were scored). (B) Cell area (µm^2) and (C) cell aspect ratio (AR) during germination on rich media (YEAS; solid lines) and synthetic media (PMG; dotted lines). The shaded area around each line represents standard error. In some instances, the standard error is thinner than the line. Note that these cell areas provided by the deep learning algorithm are slightly larger than the actual cell areas (see methods). (D) The fraction of spores manually observed to germinate on YEAS and PMG by the end of the 1,400-minute timelapse. N>50 spores per sample. ** indicates p-value < 0.01, Fisher's exact test. (E) Time of first division (when the AR of a cell exceeded 3) on YEAS and

PMG. ** indicates p-value <0.01 via ANOVA test with post-hoc t-tests for specific comparisons. N>100 spores per sample.

Figure S4. Storage temperature affects spore health. The data in A-B are the same data as those shown in Figure 1C-D but are plotted differently (individual data points rather than population averages) to more clearly illustrate how cell aspect ratio and area change over the course of germination. (A) Aspect Ratio (AR) over time of spores germinating on rich media (YEAS) at 32°C after previous storage of 3 days at 25°C or 42°C in water. (B) Two-dimensional histograms showing the AR and cell area of the entire population of spores analyzed in panel (A). Contour lines were added to help visualize concentrations of cells. (C) The starting area (μm^2) of all spores (left) or of only the successfully germinated spores analyzed in panel (A). N>50 spores per sample. ** indicates p-value < 0.01 via ANOVA test with post-hoc t-tests for specific comparisons.

Figure S5. Storage temperature affects spore health. Imaging analyses of spores stored for three days at 25°C or 42°C before being plated on media to induce germination at 32°C. Cells were imaged every 10 minutes for 1,750 minutes. (A) Timelapse microscopy of spores germinating on rich media (YEAS, left) and synthetic media (PMG, right). The yellow arrows illustrated an elongated germination phenotype. The time in minutes is displayed and the scale bars represent 10 μm. (B) Cell area (μm^2) and (C) cell aspect ratio (AR) during germination on rich media (YEAS; solid lines) and synthetic media (PMG; dotted lines). The shaded area around each line represents standard error. In some instances, the standard error is thinner than the line. Note that these cell areas provided by the deep learning algorithm are slightly larger than the actual cell areas (see methods). (D) The fraction of spores manually observed to germinate on YEAS and PMG by the end of the 1,750-minute timelapse. N>50 spores per sample. ** indicates p-value < 0.01 via Fisher's exact test. (E) The percentage of spores

displaying the indicated outgrowth phenotypes on YEAS and PMG. N>50 outgrowths per sample. ** indicates p-value < 0.01 via Fisher's exact test. (F) Time of first division (when the AR of a cell exceeded 3) on YEAS and PMG. ** indicates p-value <0.01 via ANOVA test with post-hoc t-tests for specific comparisons. N>100 spores per sample. (G) Starting area (μm^2) of all (left) or of only the successfully germinated (right) spores on YEAS and PMG. N>50 spores per sample. ** indicates p-value < 0.01 via ANOVA test with post-hoc t-tests for specific comparisons. (H) Average time of first division and (I) Average germination efficiency of spores stored for 2-4 days at 25°C (N=6) or 42°C (N=6) across all timelapses with various germination temperatures and media.

Figure S6. Storage temperature affects spore health. Imaging analyses of spores stored for 28 days at 25°C or 37°C before germination was induced at 32°C on rich media (YEAS). Cells were imaged every 10 minutes for 1,200 minutes. (A) Timelapse microscopy images of germinating spores. The time in minutes is displayed and the scale bars represent 10 μm. (B) Cell area (μm^2) and (C) cell aspect ratio (AR) during germination. The shaded area around each line represents standard error. In some instances, the standard error is thinner than the line. Note that these cell areas provided by the deep learning algorithm are slightly larger than the actual cell areas (see methods). (D) The fraction of spores manually observed to germinate by the end of the 1,200-minute timelapse. N>50 spores per sample. ** indicates p-value < 0.01, Fisher's exact test. (E) The percentage of spores displaying the indicated outgrowth phenotypes. N>50 outgrowths per sample. ** indicates p-value < 0.01, Fisher's exact test. (F) Time of first division (when the AR of a cell exceeded 3). ** indicates p-value <0.01 via ANOVA test with post-hoc t-tests for specific comparisons. N>100 spores per sample. (G) Starting area (μm^2) of all (left) or of only successfully germinated (right) spores. N>50 spores per sample. ** indicates p-value < 0.01 via ANOVA test with post-hoc t-tests for specific comparisons.

Figure S7. Storage temperature affects spore health. Imaging analyses of spores stored 28 days at 25°C or 37°C before germination was induced on rich media (YEAS) or synthetic media (PMG) at 32°C. The cells were imaged every 10 minutes for 3,000 minutes. (A) Timelapse microscopy images of cells during germination. The storage temperature and germination medium are listed to the right of each row of images. The time in minutes is displayed and the scale bars represent 10 µm. (B) Cell area (μm^2) and (C) cell aspect ratio (AR) during germination. The shaded area around each line represents standard error. In some instances, the standard error is thinner than the line. Note that these cell areas provided by the deep learning algorithm are slightly larger than the actual cell areas (see methods). (D) The fraction of spores manually observed to germinate by the end of the 3000-minute timelapse. N>50 spores per sample. ** indicates p-value < 0.01 via Fisher's exact test. (F) Time of first division (when the AR of a cell exceeded 3). ** indicates p-value <0.01 via ANOVA test with post-hoc t-tests for specific comparisons. N>100 spores per sample. (E) The percentage of spores displaying the indicated outgrowth phenotypes. N>50 outgrowths per sample. ** indicates p-value < 0.01 via Fisher's exact test.

Figure S8. Storage temperature affects spore health. Imaging analyses of spores stored for 15 days at 25°C or 37°C before germination was induced at 37°C on rich media (YEAS). Cells were imaged every 10 minutes for 1,200 minutes. (A) Timelapse microscopy images of germinating spores. The time in minutes is displayed and the scale bars represent 10 µm. (B) Cell area (μm^2) and (C) cell aspect ratio (AR) during germination. The shaded area around each line represents standard error. In some instances, the standard error is thinner than the line. Note that these cell areas provided by the deep learning algorithm are slightly larger than the actual cell areas (see methods). (D) The fraction of spores manually observed to germinate by the end of the 1,200-minute timelapse. N>50 spores per sample. ** indicates p-value < 0.01 via Fisher's exact test. (E) The percentage of spores displaying the indicated outgrowth

phenotypes. N>50 outgrowths per sample. ** indicates p-value < 0.01 via Fisher's exact test. (F) Time of first division (when the AR of a cell exceeded 3). ** indicates p-value <0.01 via ANOVA test with post-hoc t-tests for specific comparisons. N>100 spores per sample. (G) Average time of first division and (I) Average germination efficiency of spores stored for 15-35 days at 25°C (N=9) or 37°C (N=4) across all timelapses with various germination temperatures and media.

Figure S9. Storage temperature affects spore health. (A) Scanning electron microscopy images of spores stored for 4 days at 25°C (top), 4 days at 42°C (middle), and 243 days at 25°C (bottom). The inset on the 243-day old sample centers on a 'deflated' spore. Scale bars represent 1 μm. (B) Percentage of deflated and round spores observed in samples represented in panel (A). N> 50 spores. ** indicates p-value < 0.01 via Fisher's exact test. (C) Scanning transmission electron microscopy images of spores stored for 3 days at 25°C (top), 3 days at 42°C (middle), or 270 days at 25°C (bottom). Each of the insets focus on a 'dark-staining' spore, but a 'light-staining' spore is just to the left of the inset spore in the 42°C sample. Scale bars represent 1 μm. (D) Percentage of spores stored for 3 days at 25°C or 42°C, or 270 days at 25°C with light-staining or dark-staining cytoplasm. N> 50 spores. ** indicates p-value < 0.01 via Fisher's exact test.

Figure S10. Storage media significantly affects spore longevity, but not health. (A) One population of spores was split and resuspended in either 0.5% yeast extract solution without glucose (YE) or water and then stored at 4°C. Panel (B) shows a repeat of the experiment shown in (A). In both experiments, the stored spores were then sampled over time to assay the number of colony-forming units (CFUs) on rich media (YEAS), which were normalized to the time zero starting point. In (A), three replicates are shown for each storage medium. In (B), The average of 3 replicates is shown and the error bars represent standard deviations. ** indicates p value < 0.01, * indicates p value < 0.05, comparing areas under the curves via t-test. (C-G)

Imaging analyses of spores stored for 67 days at 4°C in either water or yeast extract solution before being plated on rich media to induce germination at 32°C. Cells were imaged every 10 minutes for 1,200 minutes. (C) Timelapse microscopy images of germinating spores. The time in minutes is displayed and the scale bars represent 10 μm. (D) Cell area (μm^2) and (E) cell aspect ratio (AR) during germination. The shaded area around each line represents standard error. In some instances, the standard error is thinner than the line. Note that these cell areas provided by the deep learning algorithm are slightly larger than the actual cell areas (see methods). (F) Time of first division (time when the AR of a cell exceeded 3). NS indicates not significant via ANOVA test with post-hoc t-tests for specific comparisons. N>100 spores per sample. (G) The fraction of spores manually observed to germinate by the end of the 1,200-minute timelapse. N>50 spores per sample. NS indicates not significant via Fisher's exact test.

Figure S11. Storage media significantly affects spore longevity, but not health. Imaging analyses of spores stored for 7 days at 42°C in either water or 0.5% yeast extract solution without glucose (YE) before being plated on rich media (YEAS) to induce germination at 32°C. Cells were imaged every 10 minutes for 1,200 minutes. (A) Timelapse microscopy images of germinating spores. The time in minutes is displayed and the scale bars represent 10 μm. (B) Cell area (μm^2) and (C) cell aspect ratio (AR) during germination. The shaded area around each line represents standard error. In some instances, the standard error is thinner than the line. Note that these cell areas provided by the deep learning algorithm are slightly larger than the actual cell areas (see methods). (D) Time of first division (time when the AR of a cell exceeded 3). NS indicates not significant via ANOVA test with post-hoc t-tests for specific comparisons. N>100 spores per sample. (E) The fraction of spores manually observed to germinate by the end of the 1,200-minute timelapse. N>50 spores per sample. NS indicates not significant via Fisher's exact test.

Figure S12. Storage media significantly affects spore longevity, but not health. Imaging analyses of spores stored for 58 days at 4°C in either water or 0.5% yeast extract solution without glucose (YE) before being plated on rich media (YEAS) to induce germination at 37°C. Cells were imaged every 10 minutes for 1,200 minutes. (A) Timelapse microscopy images of germinating spores. The time in minutes is displayed and the scale bars represent 10 μm. (B) Cell area (μm^2) and (C) cell aspect ratio (AR) during germination. The shaded area around each line represents standard error. In some instances, the standard error is thinner than the line. Note that these cell areas provided by the deep learning algorithm are slightly larger than the actual cell areas (see methods). (D) Time of first division (time when the AR of a cell exceeded 3). NS indicates not significant via ANOVA test with post-hoc t-tests for specific comparisons. N>100 spores per sample. (E) The fraction of spores manually observed to germinate by the end of the 1,200-minute timelapse. N>50 spores per sample. NS indicates not significant via Fisher's exact test. (F) Average time of first division and (G) Average germination efficiency of spores stored for 7-67 days in water (orange) or YE (green) at 4°C across all timelapses, including various germination temperatures and media (N=4).

Figure S13. Age affects spore health. (A) Imaging analyses of spores stored for 3, 157, or 282 days at 25°C in water before being plated on rich media (YEAS) to induce germination at 32°C. Cells were imaged every 10 minutes for 1,200 minutes. (A) Timelapse microscopy images of germinating spores. The time in minutes is displayed and the scale bars represent 10 μm. (B) Cell area (μm^2) and (C) aspect ratio (AR) during germination. The shaded area around each line represents standard error. In some instances, the standard error is thinner than the line. Note that these cell areas provided by the deep learning algorithm are slightly larger than the actual cell areas (see methods). (D) The fraction of spores manually observed to germinate by the end of the 1,200-minute timelapse. N>50 spores per sample. ** indicates p-value < 0.01 via Fisher's exact test. (E) Time of first division (time when the AR of a cell exceeded 3). ** indicates p-value

< 0.01 via ANOVA test with post-hoc t-tests for specific comparisons. N>100 spores per sample. (F) The percentage of spores displaying the indicated outgrowth phenotypes. N>50 outgrowths per sample. ** indicates p-value < 0.01 via Fisher's exact test. (G) Starting area (μm^2) of spores. N>50 spores per sample. NS indicates not significant via ANOVA test with post-hoc t-tests for specific comparisons.

Figure S14. Age affects spore health. Imaging analyses of spores stored for 7 or 176 days at 25°C in water before being plated on rich media (YEAS) in high (concentrated) or low (dilute) concentration to induce germination at 32°C. Cells were imaged every 10 minutes for 1,200 minutes. (A) Timelapse microscopy images of germinating spores. The time in minutes is displayed and the scale bars represent 10 μm. (B) Cell area (μm^2) and (C) aspect ratio (AR) during germination. The shaded area around each line represents standard error. In some instances, the standard error is thinner than the line. Note that these cell areas provided by the deep learning algorithm are slightly larger than the actual cell areas (see methods). (D) The fraction of spores manually observed to germinate by the end of the 1,200-minute timelapse. N>50 spores per sample. ** indicates p-value < 0.01 via Fisher's exact test. (E) Time of first division (time when the AR of a cell exceeded 3). ** indicates p-value < 0.01 via ANOVA test with post-hoc t-tests for specific comparisons. N>100 spores per sample.

Figure S15. Age affects spore health. Imaging analyses of spores stored for 1 or 62 days at 25°C in water before being plated on synthetic media (PMG) to induce germination at 32°C. Cells were imaged every 10 minutes for 1,200 minutes. (A) Timelapse microscopy images of germinating spores. The time in minutes is displayed and the scale bars represent 10 μm. (B) Cell area (μm^2) and (C) aspect ratio (AR) during germination. The shaded area around each line represents standard error. In some instances, the standard error is thinner than the line. Note that these cell areas provided by the deep learning algorithm are slightly larger than the actual

cell areas (see methods). (D) The fraction of spores manually observed to germinate by the end of the 1,200-minute timelapse. N>50 spores per sample. ** indicates p-value < 0.01 via Fisher's exact test. (E) Time of first division (time when the AR of a cell exceeded 3). ** indicates p-value < 0.01 via ANOVA test with post-hoc t-tests for specific comparisons. N>100 spores per sample.

Figure S16. Age affects spore health. Imaging analyses of spores stored for 1 or 85 days at 25°C in water before being plated on rich media (YEAS) to induce germination at 37°C. Cells were imaged every 10 minutes for 1,200 minutes. (A) Timelapse microscopy images of germinating spores. The time in minutes is displayed and the scale bars represent 10 μm. (B) Cell area (μm^2) and (C) aspect ratio (AR) during germination. The shaded area around each line represents standard error. In some instances, the standard error is thinner than the line. Note that these cell areas provided by the deep learning algorithm are slightly larger than the actual cell areas (see methods). (D) The fraction of spores manually observed to germinate by the end of the 1,200-minute timelapse. N>50 spores per sample. ** indicates p-value < 0.01 via Fisher's exact test. (E) Time of first division (time when the AR of a cell exceeded 3). ** indicates p-value < 0.01 via ANOVA test with post-hoc t-tests for specific comparisons. N>100 spores per sample. (F) Average time of first division and (G) Average germination efficiency on YEAS of spores stored for 1-20, 30-100, or 150-240 days in water at 25°C across all timelapses, including various germination temperatures and media (N=4).

Figure S17. Age affects asymmetry of vacuole segregation during germination. Imaging analyses of vacuole segregation in FM4-64-stained spores previously stored at 25°C in water and then germinated on rich media (YEAS) at 32°C. The cells were imaged every 10 minutes for 20 hours. (A, C) Timelapse microscopy images of FM4-64-stained spores aged for 1 or 34 days (A) and 3 or 169 days (C). The brightness and contrast are not the same for all images but were adjusted so the spore bodies appeared to have similar levels of signal. Scale bars

represent 10 μm. (B, D) Percentage of the total FM4-64 signal retained in the spore-body verses the germ tube outgrowth for spores previously aged for 1 or 34 days (B) and 3 or 169 days (D). ** indicates p-value <0.01 via Fisher's exact test. N> 30 spores.

Figure S18. Age affects asymmetry of vacuole segregation during germination. Imaging analyses of vacuole segregation in FM4-64-stained spores previously stored in water for 5 or 173 days at 25°C, or for 5 days at 42°C. (A) Timelapse microscopy images of FM4-64-stained spores germinating on rich media (YEAS) at 32°C. The brightness and contrast are not the same for all images but were adjusted so the spore bodies appeared to have similar levels of signal. Scale bars represent 10 μm. (B) Percentage of the total FM4-64 signal retained in the spore-body verses the germ tube outgrowth. ** indicates p-value <0.01 via Fisher's exact test. N> 30 spores

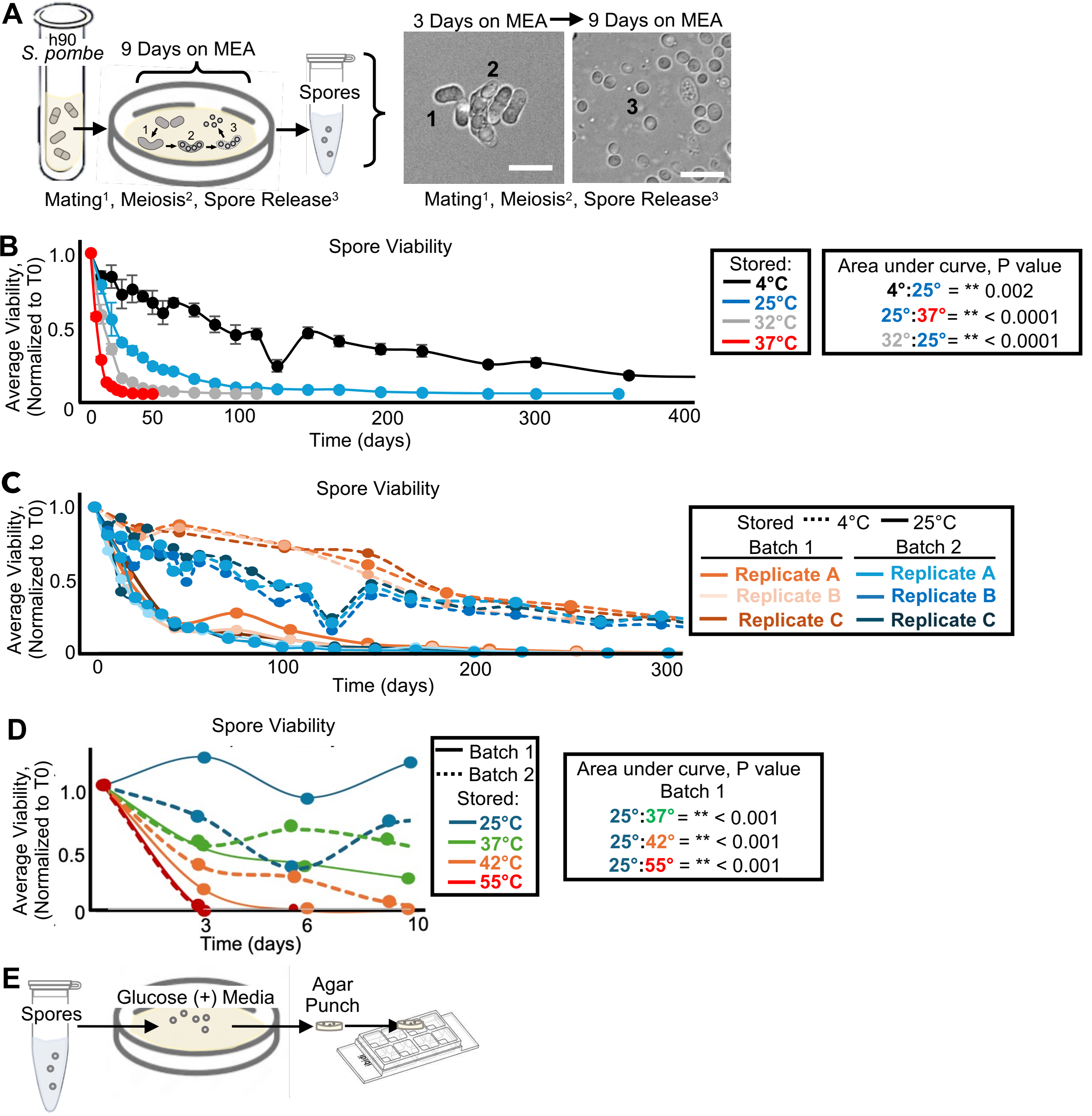
A
h90
S. pombe
9 Days on MEA
Spores
Mating¹, Meiosis², Spore Release³
3 Days on MEA → 9 Days on MEA
Mating¹, Meiosis², Spore Release³
B
Spore Viability
Average Viability, (Normalized to T0)
Time (days)
Stored:
4°C
25°C
32°C
37°C
Area under curve, P value
4°:25° = ** 0.002
25°:37°= ** < 0.0001
32°:25°= ** < 0.0001
C
Spore Viability
Average Viability, (Normalized to T0)
Time (days)
Stored 4°C 25°C
Batch 1
Batch 2
Replicate A
Replicate B
Replicate C
D
Spore Viability
Average Viability, (Normalized to T0)
Time (days)
Batch 1
Batch 2
Stored:
25°C
37°C
42°C
55°C
Area under curve, P value
Batch 1
25°:37° = ** < 0.001
25°:42° = ** < 0.001
25°:55° = ** < 0.001
E
Spores
Glucose (+) Media
Agar
Punch

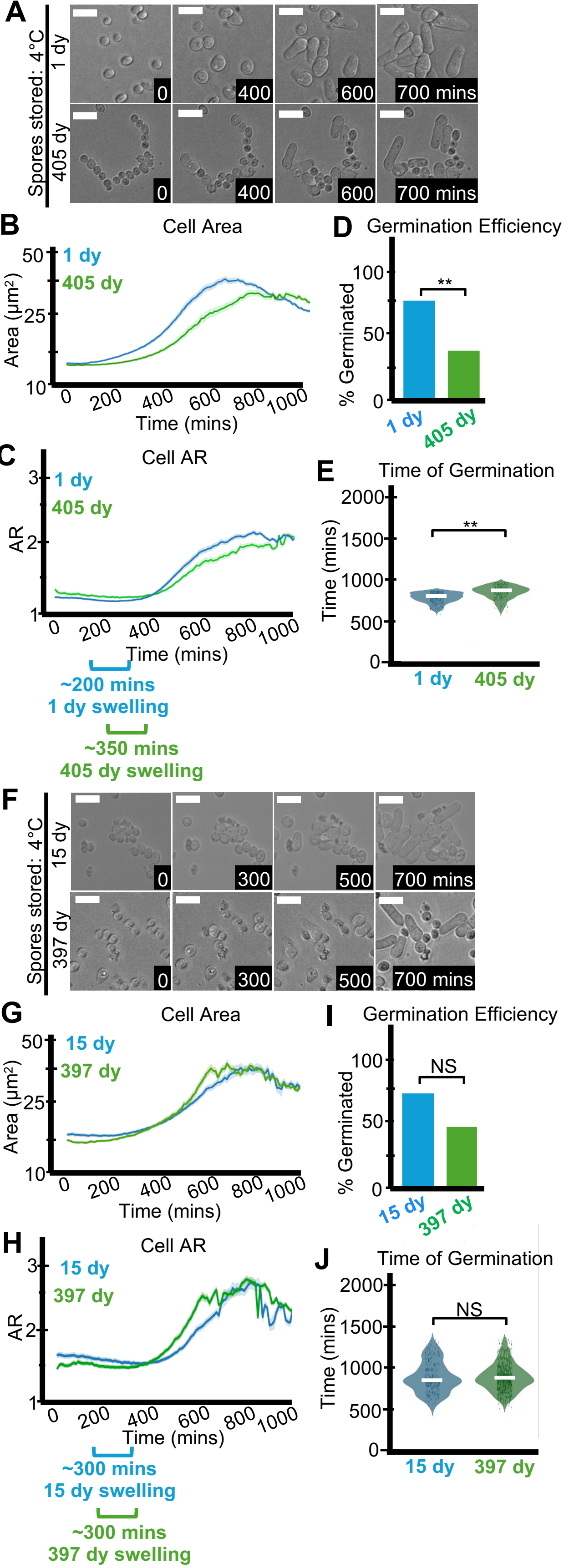
A
Spores stored: 4°C
1 dy
405 dy
0
400
600
700 mins
B
Cell Area
1 dy
405 dy
Area (µm²)
Time (mins)
C
Cell AR
1 dy
405 dy
AR
Time (mins)
~200 mins
1 dy swelling
~350 mins
405 dy swelling
D
Germination Efficiency
% Germinated
**
1 dy
405 dy
E
Time of Germination
Time (mins)
**
1 dy
405 dy
F
Spores stored: 4°C
15 dy
397 dy
0
300
500
700 mins
G
Cell Area
15 dy
397 dy
Area (µm²)
Time (mins)
H
Cell AR
15 dy
397 dy
AR
Time (mins)
~300 mins
15 dy swelling
~300 mins
397 dy swelling
I
Germination Efficiency
% Germinated
NS
15 dy
397 dy
J
Time of Germination
Time (mins)
NS
15 dy
397 dy

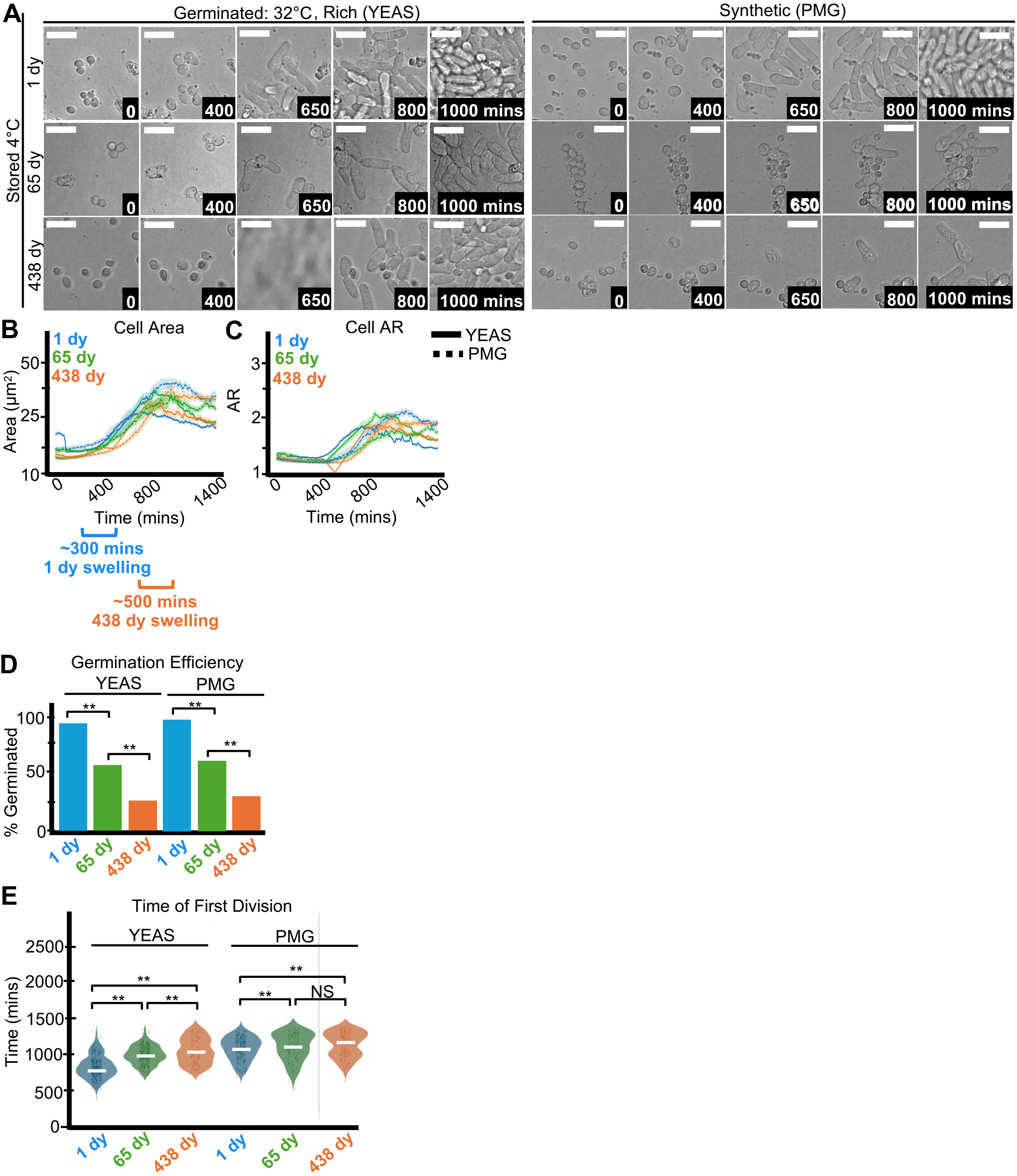
A
Germinated: 32°C, Rich (YEAS)
Synthetic (PMG)
Stored 4°C
1 dy
65 dy
438 dy
0
400
650
800
1000 mins
B
Cell Area
1 dy
65 dy
438 dy
Area (µm²)
Time (mins)
~300 mins
1 dy swelling
~500 mins
438 dy swelling
C
Cell AR
YEAS
PMG
AR
Time (mins)
D
Germination Efficiency
YEAS
PMG
% Germinated
E
Time of First Division
YEAS
PMG
NS
Time (mins)

S3

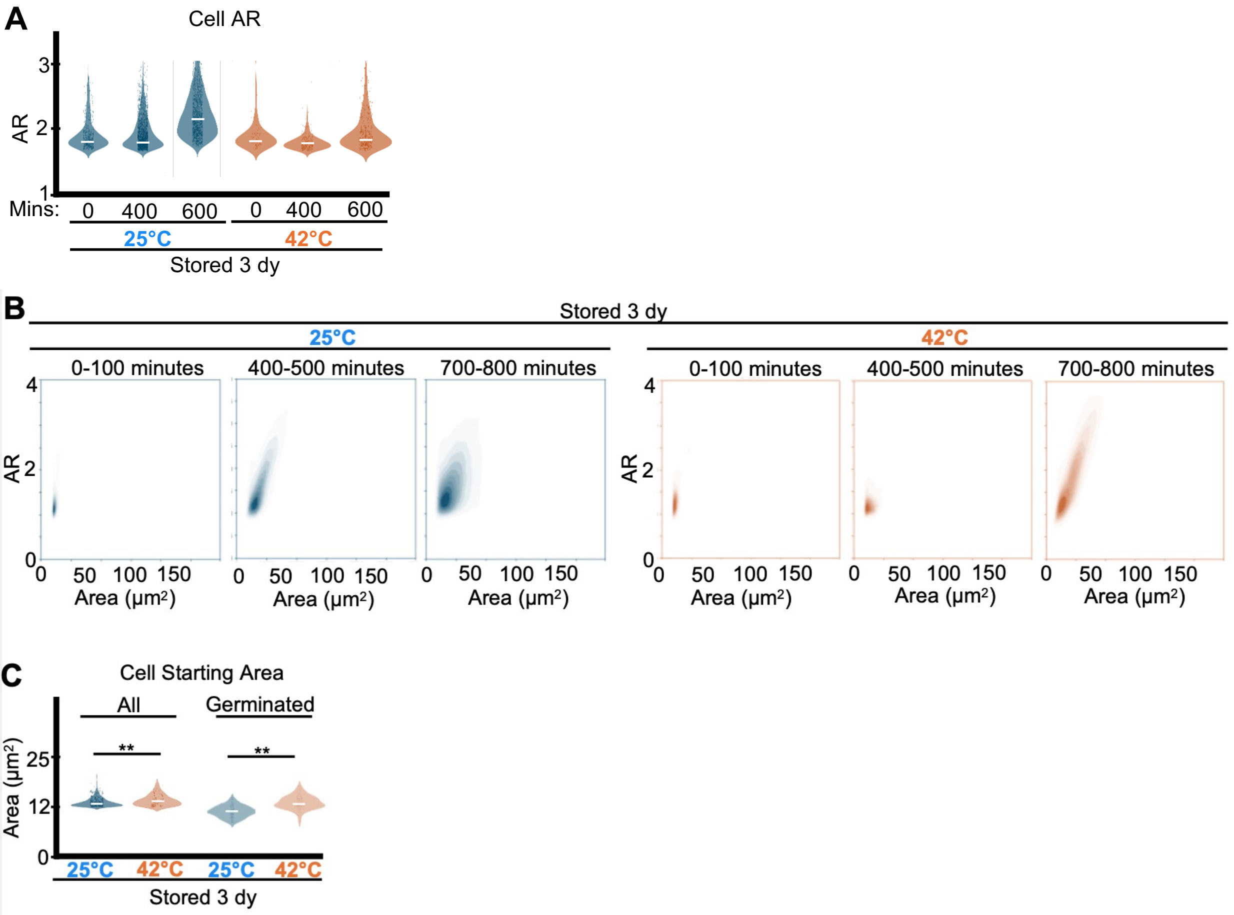

A
Cell AR
AR
3
2
1
Mins: 0 400 600 0 400 600
25°C
42°C
Stored 3 dy
B
Stored 3 dy
25°C
42°C
0-100 minutes
400-500 minutes
700-800 minutes
AR
4
2
0
0 50 100 150
Area (µm2)
C
Cell Starting Area
All
Germinated
**
Area (µm2)
25
12
0
25°C 42°C 25°C 42°C
Stored 3 dy

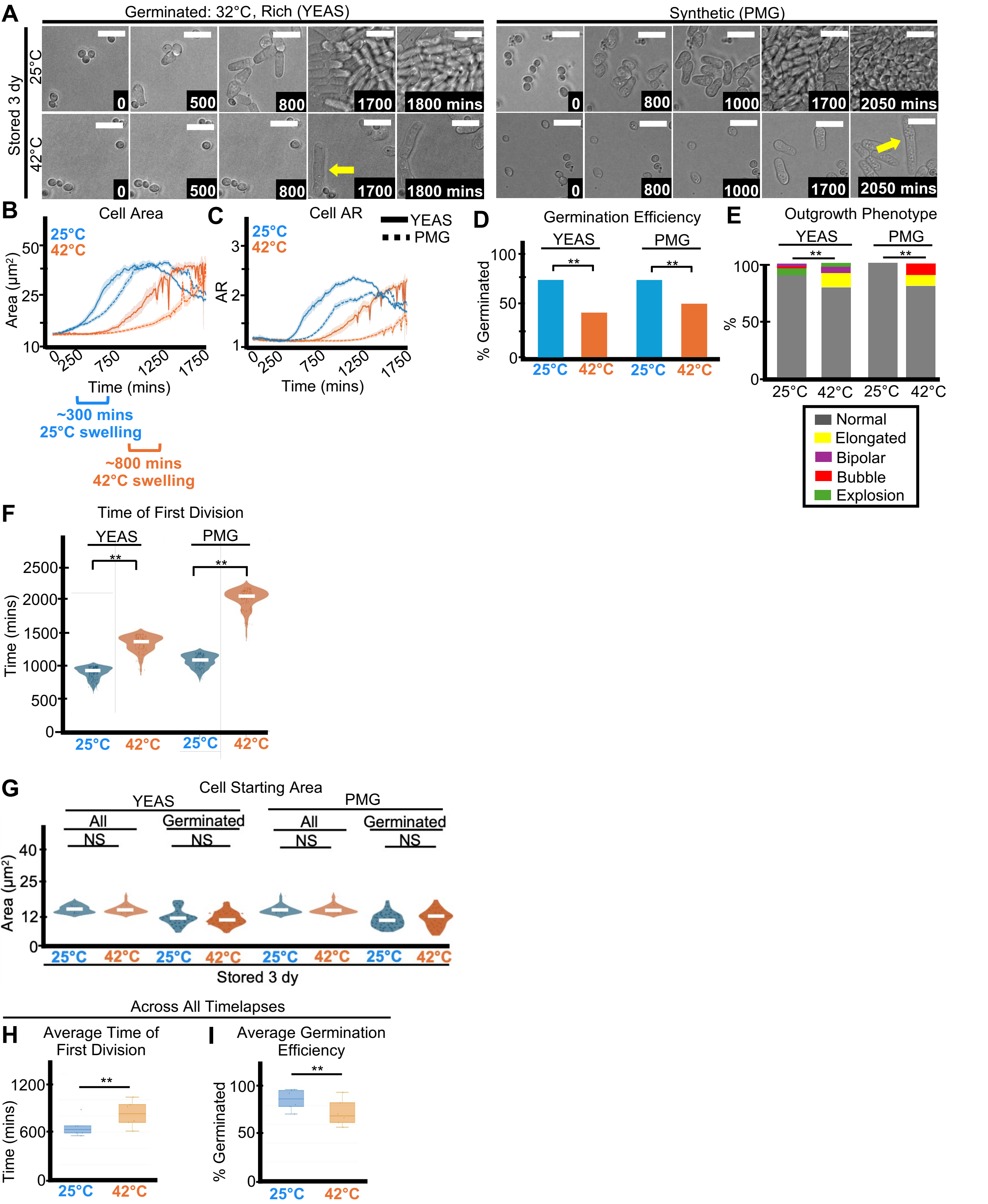

A
Germinated: 32°C, Rich (YEAS)
Synthetic (PMG)
Stored 3 dy
25°C
42°C
0
500
800
1700
1800 mins
1000
2050 mins
B
Cell Area
Area (µm²)
25°C
42°C
Time (mins)
~300 mins
25°C swelling
~800 mins
42°C swelling
C
Cell AR
AR
YEAS
PMG
Time (mins)
D
Germination Efficiency
% Germinated
YEAS
PMG
**
E
Outgrowth Phenotype
%
Normal
Elongated
Bipolar
Bubble
Explosion
F
Time of First Division
Time (mins)
G
Cell Starting Area
All
Germinated
NS
Area (µm²)
Stored 3 dy
Across All Timelapses
H
Average Time of First Division
Time (mins)
I
Average Germination Efficiency
% Germinated

S5

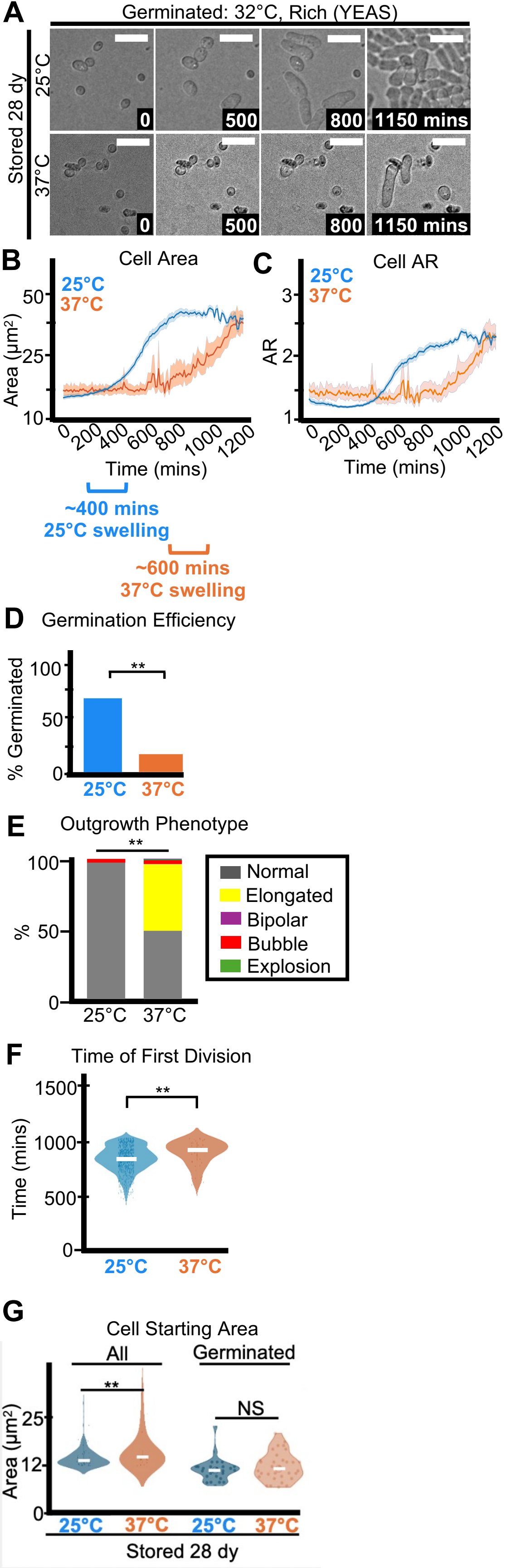
A
Germinated: 32°C, Rich (YEAS)
Stored 28 dy
25°C
37°C
0
500
800
1150 mins
B
Cell Area
25°C
37°C
Area (µm²)
50
25
10
0
200
400
600
800
1000
1200
Time (mins)
~400 mins
25°C swelling
~600 mins
37°C swelling
C
Cell AR
AR
3
2
1
Time (mins)
D
Germination Efficiency
% Germinated
100
50
0
**
25°C
37°C
E
Outgrowth Phenotype
**
%
100
50
0
Normal
Elongated
Bipolar
Bubble
Explosion
F
Time of First Division
Time (mins)
1500
1000
500
0
**
G
Cell Starting Area
All
Germinated
**
NS
Area (µm²)
25
12
0
Stored 28 dy

S6

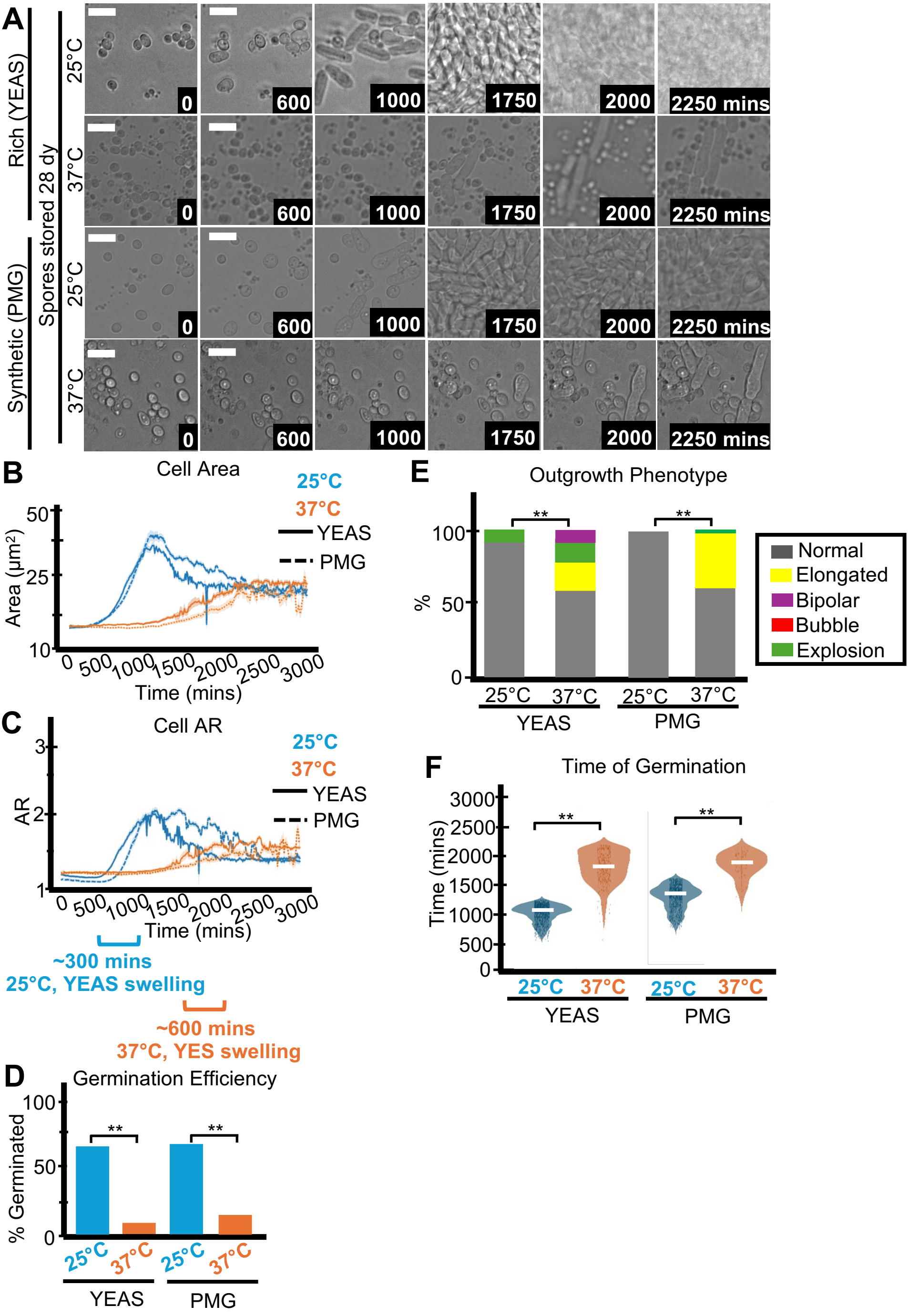
A
Rich (YEAS)
Synthetic (PMG)
Spores stored 28 dy
25°C
37°C
0
600
1000
1750
2000
2250 mins
B
Cell Area
25°C
37°C
YEAS
PMG
Area (µm²)
Time (mins)
C
Cell AR
AR
~300 mins
25°C, YEAS swelling
~600 mins
37°C, YES swelling
D
Germination Efficiency
% Germinated
E
Outgrowth Phenotype
%
Normal
Elongated
Bipolar
Bubble
Explosion
YEAS
PMG
F
Time of Germination
Time (mins)

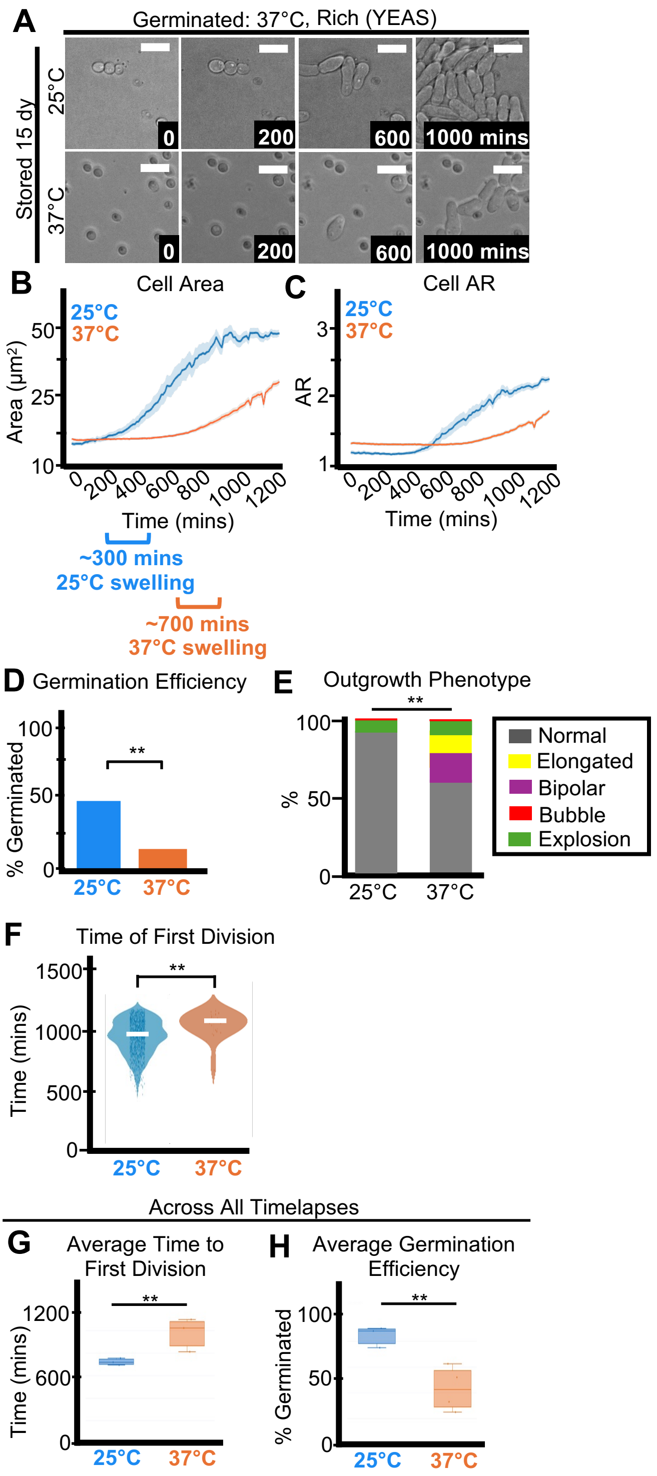

A
Germinated: 37°C, Rich (YEAS)
Stored 15 dy
25°C
37°C
0
200
600
1000 mins
B
Cell Area
Area (µm²)
25°C
37°C
Time (mins)
~300 mins
25°C swelling
~700 mins
37°C swelling
C
Cell AR
AR
Time (mins)
D
Germination Efficiency
% Germinated
**
E
Outgrowth Phenotype
%
Normal
Elongated
Bipolar
Bubble
Explosion
F
Time of First Division
Time (mins)
Across All Timelapses
G
Average Time to First Division
Time (mins)
H
Average Germination Efficiency
% Germinated

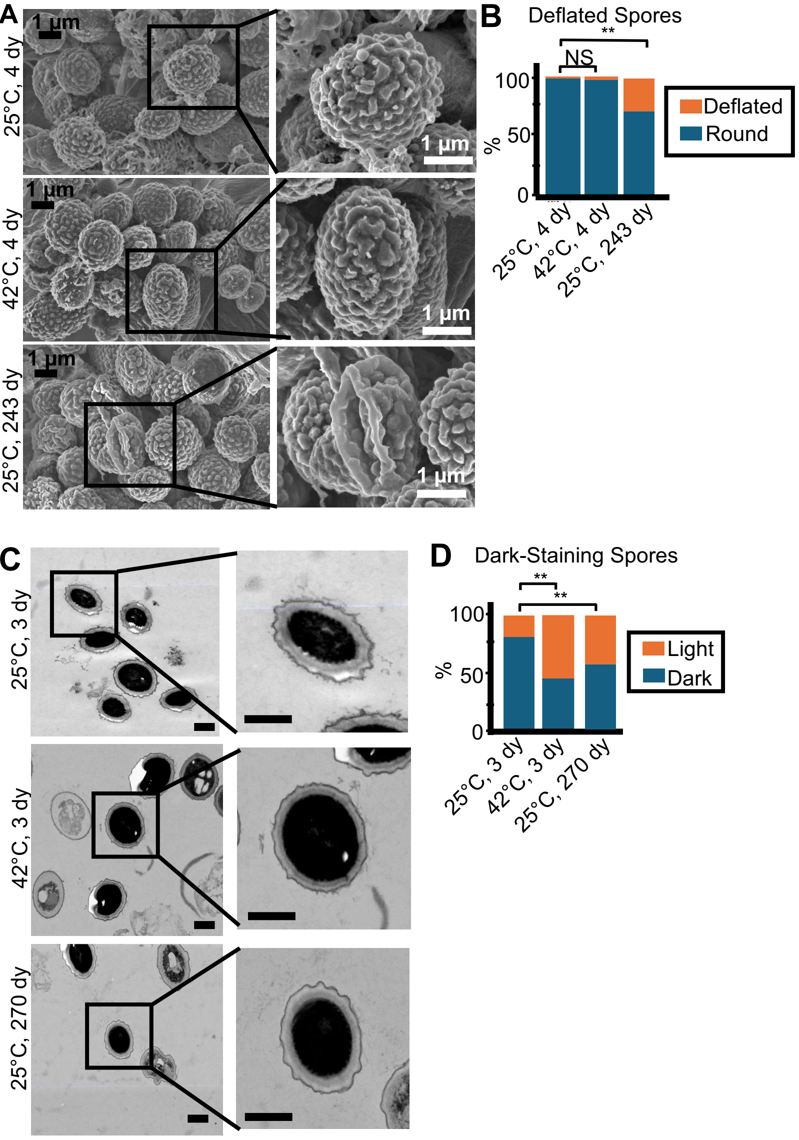

A
25°C, 4 dy
42°C, 4 dy
25°C, 243 dy
1 µm
B
Deflated Spores
NS
**
%
100
50
0
Deflated
Round
25°C, 4 dy
42°C, 4 dy
25°C, 243 dy
C
25°C, 3 dy
42°C, 3 dy
25°C, 270 dy
D
Dark-Staining Spores
**
**
Light
Dark

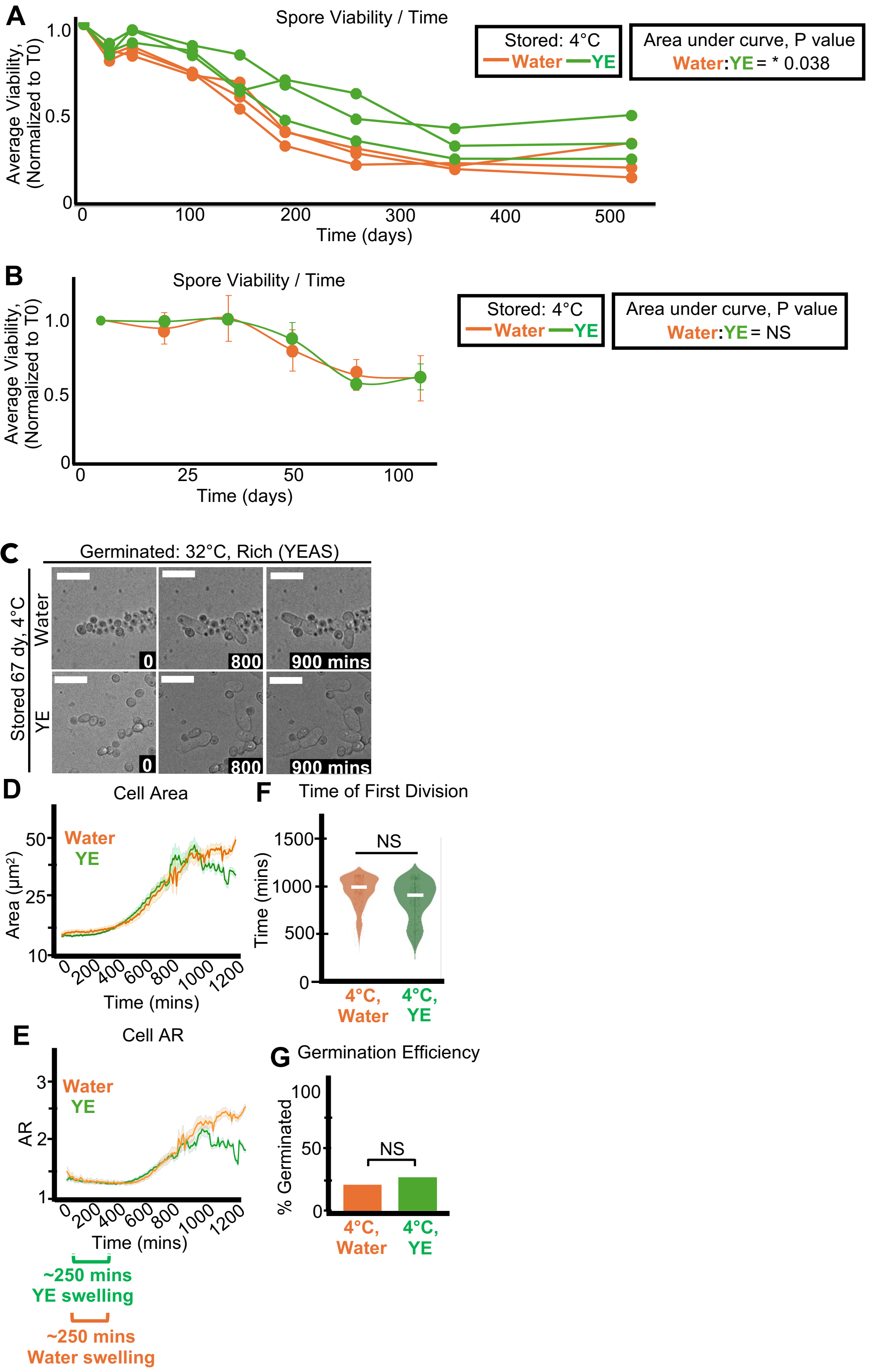
A
Spore Viability / Time
Stored: 4°C
Water
YE
Area under curve, P value
Water:YE = * 0.038
Average Viability, (Normalized to T0)
Time (days)
B
Spore Viability / Time
Stored: 4°C
Water
YE
Area under curve, P value
Water:YE = NS
Average Viability, (Normalized to T0)
Time (days)
C
Germinated: 32°C, Rich (YEAS)
Stored 67 dy, 4°C
Water
YE
0
800
900 mins
D
Cell Area
Water
YE
Area (µm²)
Time (mins)
F
Time of First Division
NS
Time (mins)
4°C, Water
4°C, YE
E
Cell AR
Water
YE
AR
Time (mins)
~250 mins YE swelling
~250 mins Water swelling
G
Germination Efficiency
NS
% Germinated
4°C, Water
4°C, YE

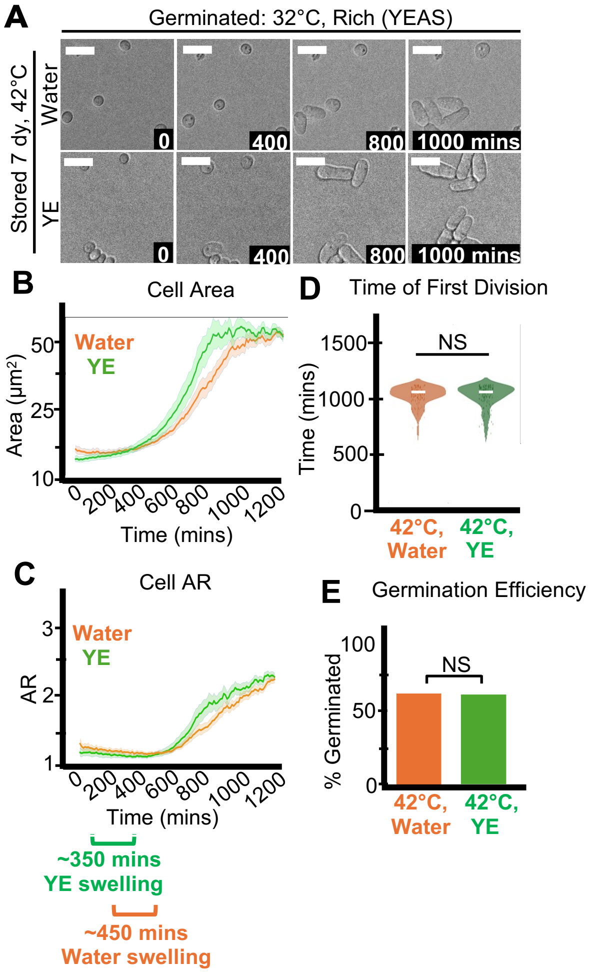
A
Germinated: 32°C, Rich (YEAS)
Stored 7 dy, 42°C
Water
YE
0
400
800
1000 mins
B
Cell Area
Water
YE
Area (μm²)
Time (mins)
C
Cell AR
AR
~350 mins
YE swelling
~450 mins
Water swelling
D
Time of First Division
Time (mins)
NS
42°C, Water
42°C, YE
E
Germination Efficiency
% Germinated
NS

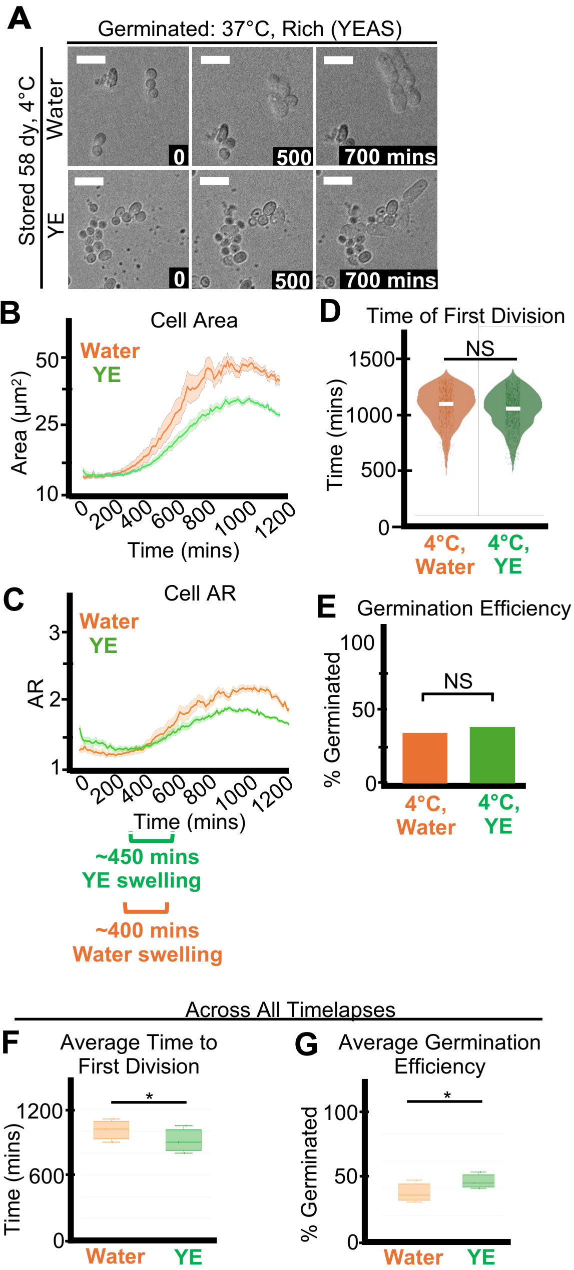
A
Germinated: 37°C, Rich (YEAS)
Stored 58 dy, 4°C
Water
YE
0
500
700 mins
B
Cell Area
Water
YE
Area (μm²)
50
25
10
0
200
400
600
800
1000
1200
Time (mins)
D
Time of First Division
NS
Time (mins)
1500
1000
500
0
4°C, Water
4°C, YE
C
Cell AR
Water
YE
AR
3
2
1
Time (mins)
~450 mins
YE swelling
~400 mins
Water swelling
E
Germination Efficiency
% Germinated
100
50
0
NS
4°C, Water
4°C, YE
Across All Timelapses
F
Average Time to First Division
Time (mins)
1200
600
0
*
Water
YE
G
Average Germination Efficiency
% Germinated
100
50
0
*
Water
YE

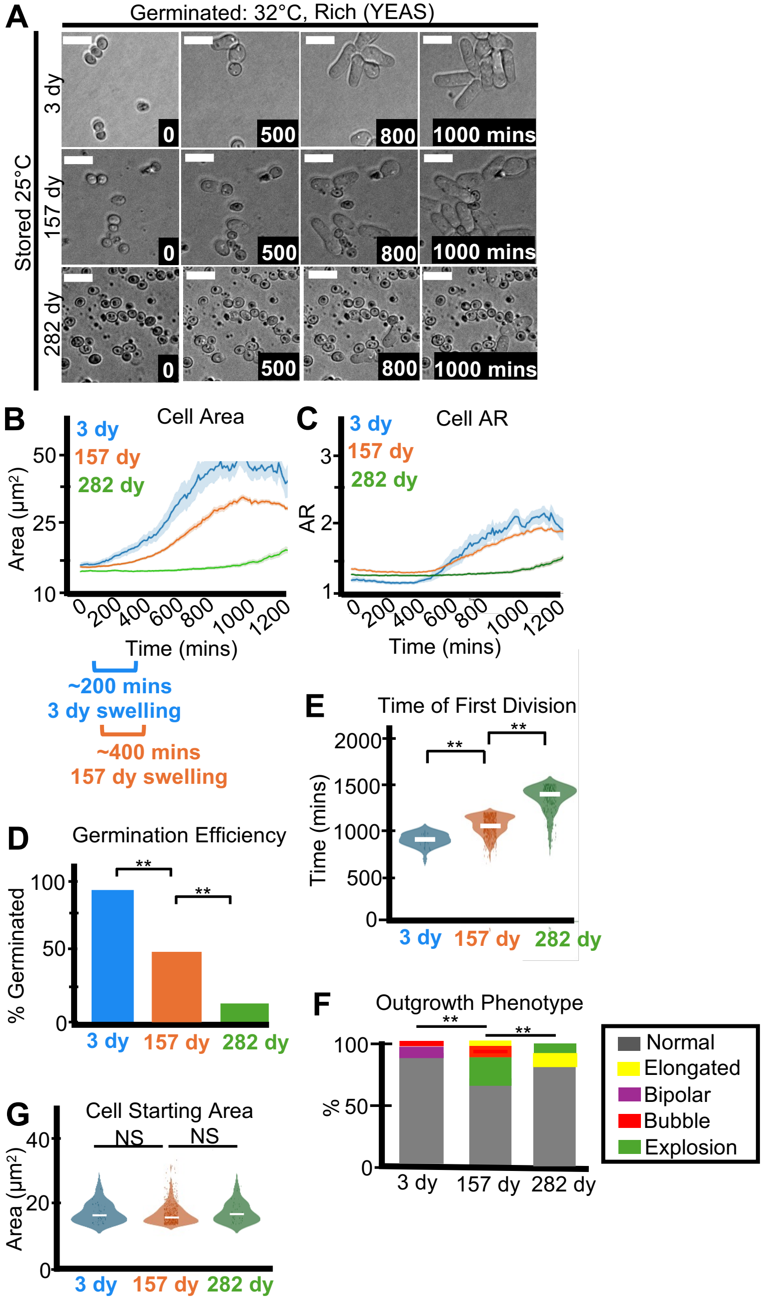
A
Germinated: 32°C, Rich (YEAS)
Stored 25°C
3 dy
157 dy
282 dy
0
500
800
1000 mins
B
Cell Area
3 dy
157 dy
282 dy
Area (μm²)
Time (mins)
~200 mins
3 dy swelling
~400 mins
157 dy swelling
C
Cell AR
AR
Time (mins)
D
Germination Efficiency
% Germinated
**
E
Time of First Division
Time (mins)
F
Outgrowth Phenotype
%
Normal
Elongated
Bipolar
Bubble
Explosion
G
Cell Starting Area
NS
Area (μm²)

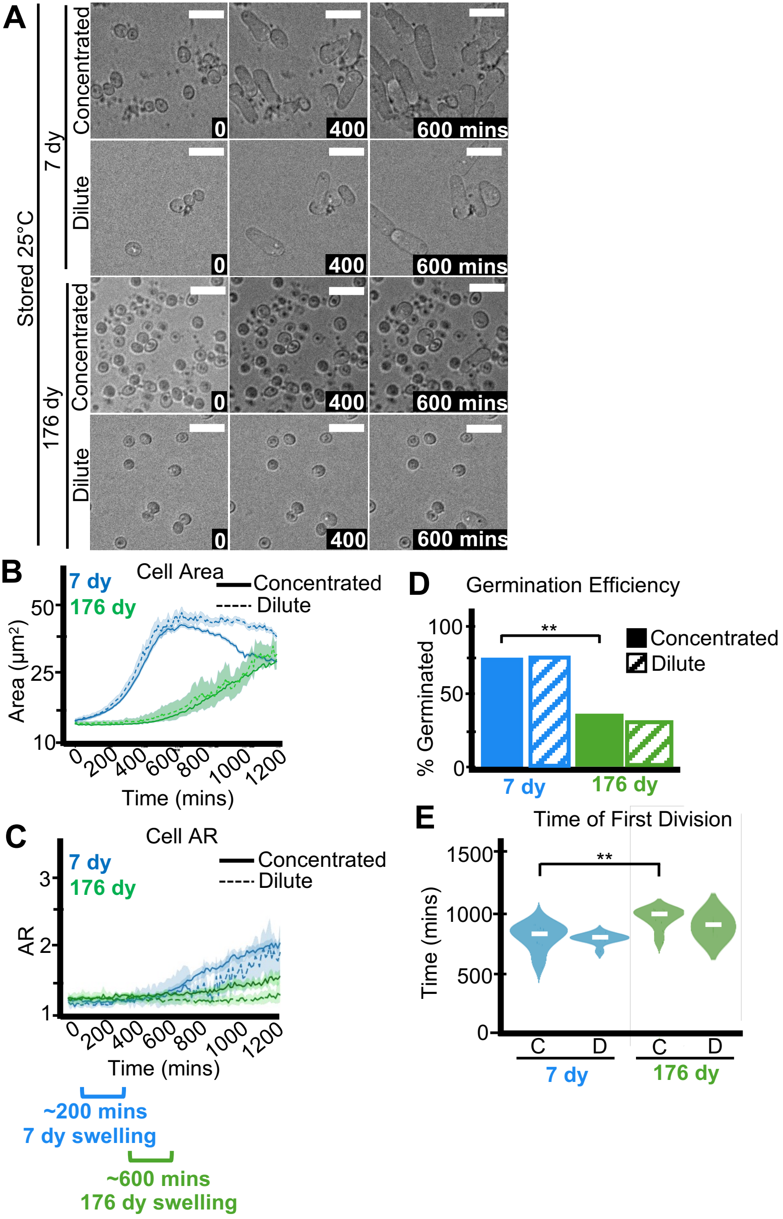

A
Stored 25°C
7 dy
176 dy
Concentrated
Dilute
0
400
600 mins
B
Cell Area
7 dy
176 dy
Concentrated
Dilute
Area (μm²)
Time (mins)
C
Cell AR
AR
~200 mins
7 dy swelling
~600 mins
176 dy swelling
D
Germination Efficiency
% Germinated
**
E
Time of First Division
Time (mins)
C
D

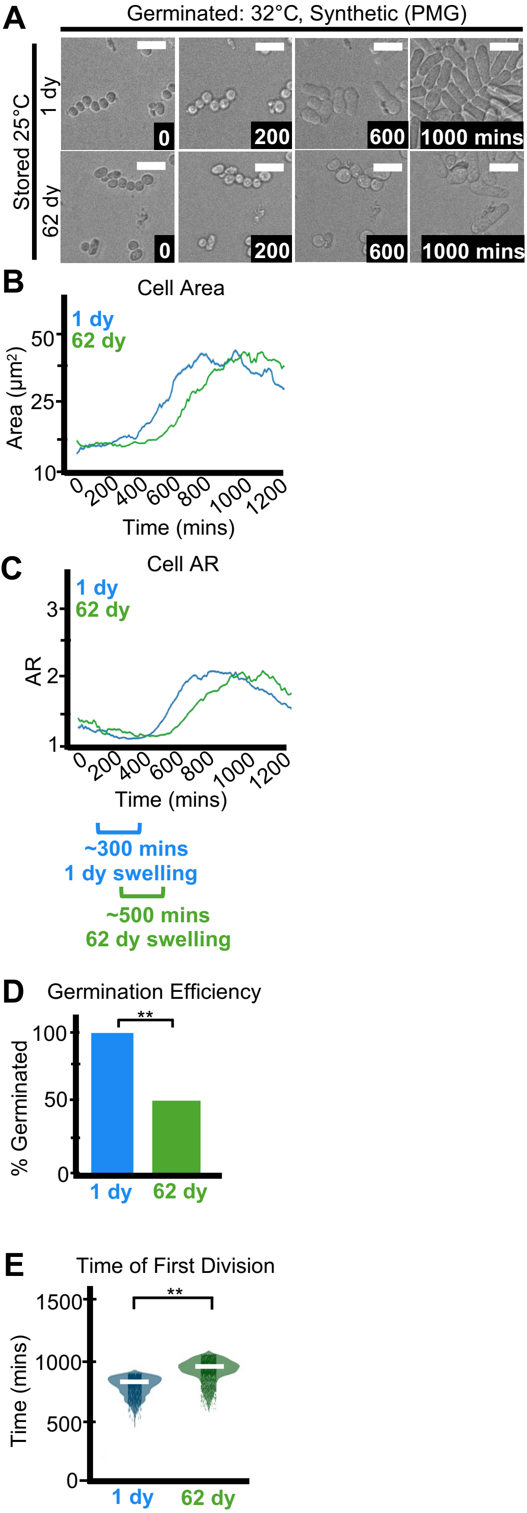
A
Germinated: 32°C, Synthetic (PMG)
Stored 25°C
1 dy
62 dy
0
200
600
1000 mins
B
Cell Area
1 dy
62 dy
Area (μm²)
Time (mins)
C
Cell AR
1 dy
62 dy
AR
Time (mins)
~300 mins
1 dy swelling
~500 mins
62 dy swelling
D
Germination Efficiency
% Germinated
**
1 dy
62 dy
E
Time of First Division
Time (mins)
**
1 dy
62 dy

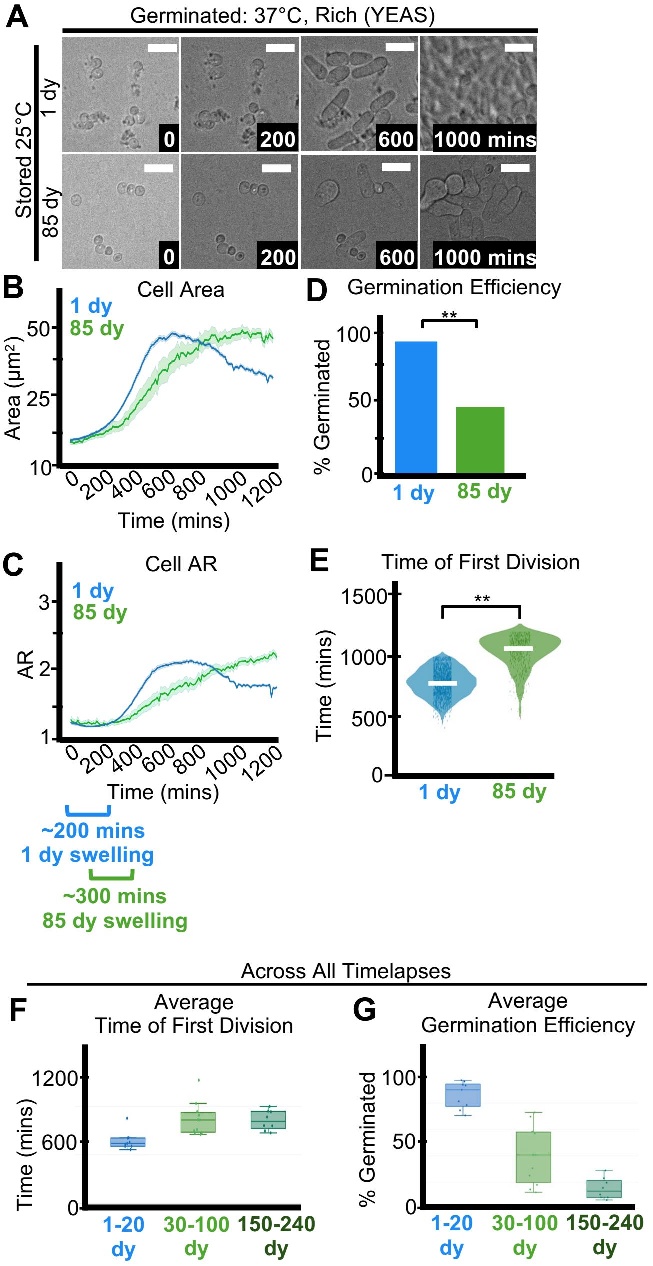
A
Germinated: 37°C, Rich (YEAS)
Stored 25°C
1 dy
85 dy
0
200
600
1000 mins
B
Cell Area
1 dy
85 dy
Area (µm²)
50
25
10
Time (mins)
0
200
400
600
800
1000
1200
C
Cell AR
1 dy
85 dy
AR
3
2
1
Time (mins)
~200 mins
1 dy swelling
~300 mins
85 dy swelling
D
Germination Efficiency
% Germinated
100
50
0
**
1 dy
85 dy
E
Time of First Division
Time (mins)
1500
1000
500
0
**
1 dy
85 dy
Across All Timelapses
F
Average
Time of First Division
Time (mins)
1200
600
0
1-20 dy
30-100 dy
150-240 dy
G
Average
Germination Efficiency
% Germinated
100
50
0
1-20 dy
30-100 dy
150-240 dy

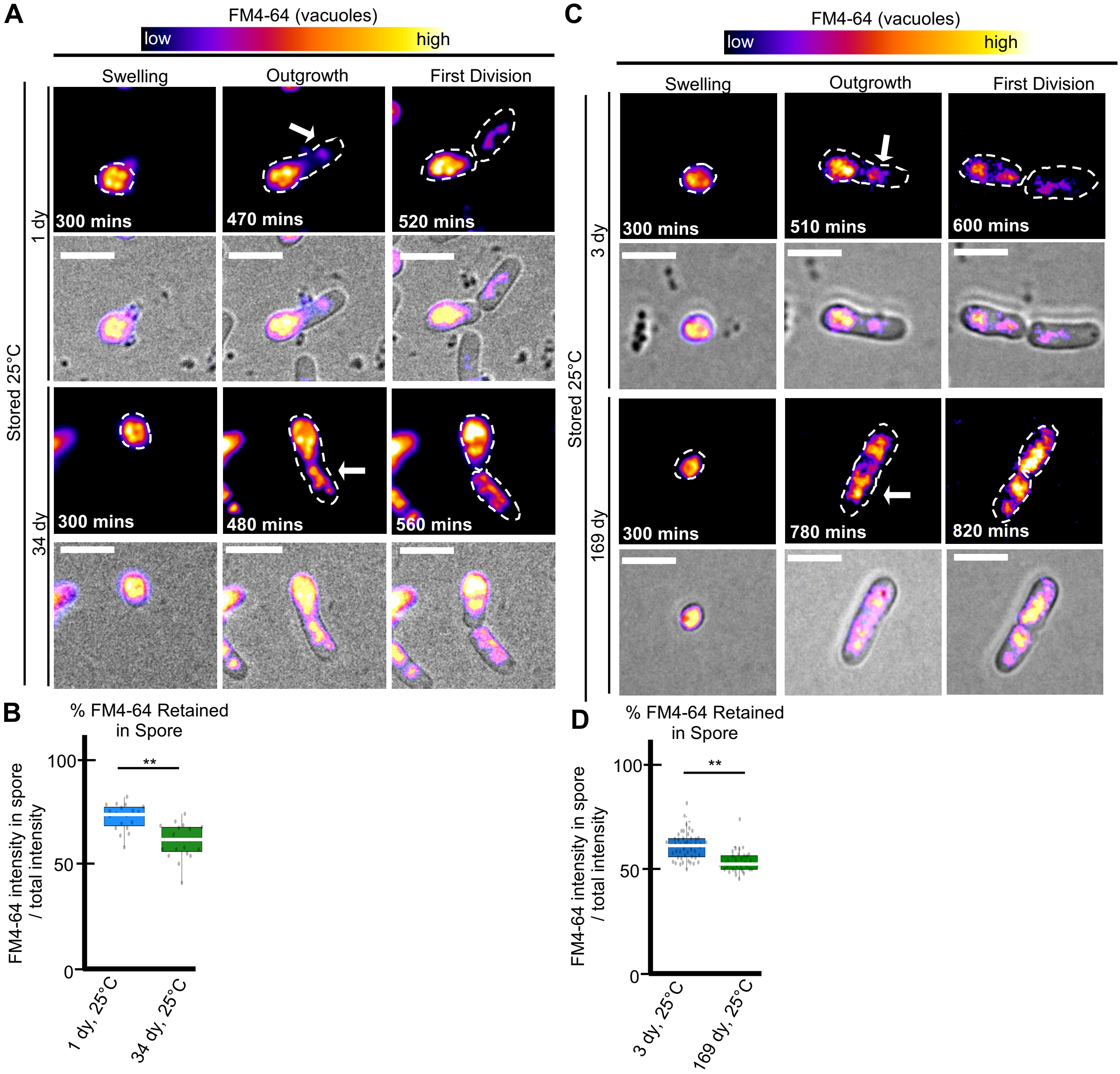
A
FM4-64 (vacuoles)
low
high
Swelling
Outgrowth
First Division
Stored 25°C
1 dy
300 mins
470 mins
520 mins
34 dy
300 mins
480 mins
560 mins
B
% FM4-64 Retained in Spore
FM4-64 intensity in spore / total intensity
100
50
0
**
1 dy, 25°C
34 dy, 25°C
C
FM4-64 (vacuoles)
low
high
Swelling
Outgrowth
First Division
Stored 25°C
3 dy
300 mins
510 mins
600 mins
169 dy
300 mins
780 mins
820 mins
D
% FM4-64 Retained in Spore
FM4-64 intensity in spore / total intensity
100
50
0
**
3 dy, 25°C
169 dy, 25°C

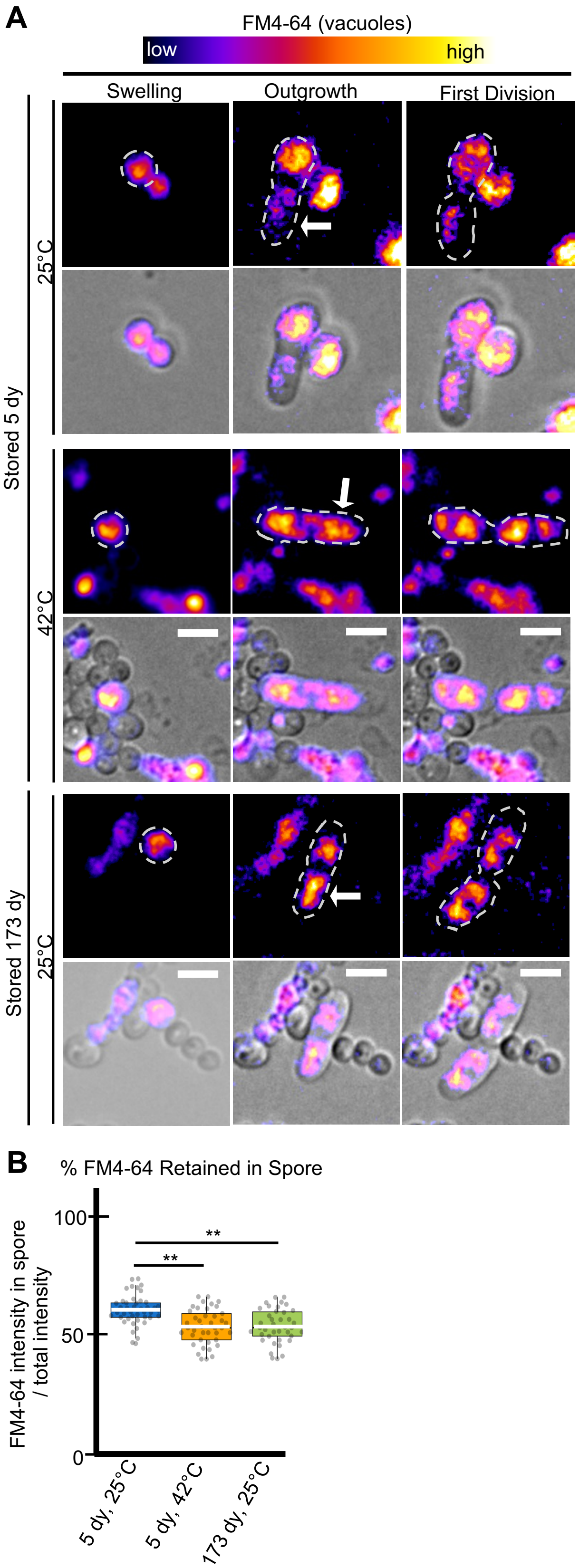
A
FM4-64 (vacuoles)
low
high
Swelling
Outgrowth
First Division
Stored 5 dy
25°C
42°C
Stored 173 dy
25°C
B
% FM4-64 Retained in Spore
FM4-64 intensity in spore / total intensity
100
50
0
**
**
5 dy, 25°C
5 dy, 42°C
173 dy, 25°C
